# Joint Target-Message Reception of Constant-Envelope CPM for Joint Radar and Communications

**DOI:** 10.3390/s26175628

**Published:** 2026-09-04

**Authors:** Jie Xu, Guangxin Wu, Jianan Liu

**Affiliations:** Nanjing Research Institute of Electronics Technology, Nanjing 210039, China; jackxu8@163.com (J.X.); 13881514641@139.com (G.W.)

**Keywords:** joint radar–communication, integrated sensing and communication, continuous phase modulation, constant-envelope waveform, joint target-message reception, random-message ambiguity function, bistatic radar

## Abstract

This paper presents a likelihood-consistent joint target-message receiver for a strictly constant-envelope continuous phase modulation (CPM) waveform in pulsed phased-array joint radar–communication (JRC). Station A performs monostatic detection with the transmitted message, while Station B performs bistatic detection and communication with an available, degraded, or absent target-independent reference path. The target-scattered message is included only under the target-present hypothesis. A log-domain 16-state recursion evaluates the exact finite-alphabet CPM message marginal over the complete 46+2-symbol, 92-bit frame and is verified against direct enumeration on a six-bit unit frame to floating-point precision. At a 10 dB reference-path SNR, marginalization increases detection probability relative to single-path max-log reconstruction by 4.67 percentage points (95% paired interval 1.33–8.00) and 8.33 percentage points (4.33–12.33) at target-path SNRs of 10 and 14 dB, respectively; the corresponding joint-success differences are zero. Across 2048 ordered error events, the directed conditional distance has a Spearman correlation of −0.970 with empirical pairwise error probability. The CPM parameterization guarantees a constant active-pulse envelope at every array element, whereas constrained common-phase selection provides only a secondary adjustment of message-dependent ambiguity sidelobes. System-level experiments in a coastal setting use independent 92-bit messages and matched transmission resources for the proposed CPM waveform and RRC-QPSK. Both waveforms recover all evaluated direct-path payloads, showing that the proposed constant-envelope waveform preserves communication reliability at the tested operating point. The same records demonstrate geometry-consistent monostatic and bistatic target recovery, while finite-scatterer tests define the boundary of the single-scattering-center model.

## 1. Introduction

Joint radar–communication (JRC), also termed dual-functional radar–communication (DFRC), is the radar-oriented form of integrated sensing and communication (ISAC) in which target and communication information are obtained from a shared radio-frequency emission. The common-waveform setting creates a statistical coupling that is absent when the two functions use separate signals. The communication message changes the phase trajectory of the transmitted waveform and therefore changes the target-echo template, ambiguity sidelobes, and message-conditioned likelihood. A receiver that does not know the realized message must account for this uncertainty rather than replacing the random waveform by a single deterministic template.

Information-theoretic studies characterize random-signal limits and radar waveform metrics [1,2,3,4,5,6,7,8,9,10,11]. Existing JRC waveform designs exploit spatial precoding, coordinated or hardware-impaired beamforming, frequency hopping, index modulation, and multicarrier signaling [12,13,14,15,16,17,18,19,20,21,22,23,24,25,26,27,28].These approaches provide spatial or waveform degrees of freedom, but their receiver assumptions do not directly address finite-state CPM message uncertainty when the target-independent reference path is degraded or absent. CPM-PARC supports saturation-efficient pulsed transmission [29], and reduced-complexity CPM communication receivers exploit the modulation trellis [30], while random-waveform and trellis-shaping methods regulate ambiguity or sidelobe statistics [15,31]; their primary objective is waveform control rather than target-hypothesis-dependent message marginalization. Data-aided and random-payload bistatic receivers use pilots, reconstructed payloads, iterative estimation, or generalized likelihoods [32,33,34,35,36,37], whereas the present model retains the CPM state memory and includes the target-scattered message only under the target-present hypothesis. The resulting gap is a strictly constant-envelope CPM waveform and a likelihood-consistent receiver that remains valid as the target-independent reference changes from available to absent.

Conditional value-at-risk (CVaR) has been used for random radar cross-section and uncertain target location [38,39]. Here it is only a design functional for the upper tail of message-induced ambiguity-sidelobe loss; detection, estimation, spectral occupancy, and communication performance are evaluated separately.

This work focuses on likelihood-consistent reception under changing reference-path conditions and on a compatible strictly constant-envelope CPM signal. Spatial transmit and receive beamforming are treated as fixed array operations; the design variable is the common baseband phase waveform. The four supported operating conditions are stable target-independent communication, target-scattered communication, monostatic detection at Station A, and bistatic detection at Station B. The principal methodological contributions are as follows.

(1) A hypothesis-consistent model is formulated for reference-assisted and reference-free reception. The target-independent reference has the same statistical role under both target hypotheses, whereas the target-scattered message is present only under the target-present hypothesis.
(2) A full-frame log-domain finite-state receiver evaluates the CPM message marginal jointly with target delay, Doppler, and framewise complex gain. The same recursion covers strong, finite, and zero reference SNR; a state-mass pruning rule is assessed as an approximation rather than assumed to provide a computational gain.
(3) A directed conditional event distance is derived from the joint likelihood after profiling the competing target parameters. It separates reference- and target-branch contributions under conditional independence, reduces continuously to the target branch as the reference vanishes, and is validated on 2048 ordered events as a local error-ordering diagnostic.
(4) The receiver is paired with a per-element constant-envelope CPM waveform. A constrained common-phase selection limits message-dependent sidelobe risk while preserving GMI and the spectral constraint; its modest effect is reported as a secondary waveform-design result and not as the source of the receiver behavior.

The remainder of the paper is organized as follows. Section 2 formulates the path and likelihood models. Section 3 presents the CPM waveform, constrained random-message design, and event-distance analysis. Section 4 develops the joint receiver. Section 5 reports waveform, full-frame receiver, model-applicability, and system-level results, followed by a discussion and conclusions in Section 6 and Section 7.

## 2. System Model and Joint Likelihood

### 2.1. Array Constraint and Propagation Model

Let m∈M denote the message carried by one pulse. The common discrete-time baseband waveform is

(1) $$x_{m}\left[n\right]=e^{j\Phi_{m}\left[n\right]},\;\left|x_{m}\left[n\right]\right|=1,\;0\leq n<N.$$

Transmit element *q* applies a fixed equal-modulus spatial weight,

(2) $$s_{q,m}\left[n\right]=P_{q}e^{j\phi_{q}}x_{m}\left[n\right],\;\left|s_{q,m}\left[n\right]\right|=P_{q}.$$

Thus, every element has constant active-pulse power. Pulse gating creates the interpulse zero interval and is excluded from the intrapulse PAPR definition. Digital beamforming is a conventional array operation and is not optimized in this study; fixed transmit and receive responses are absorbed into equivalent baseband propagation operators.

At Station B, the target-independent reference operator $G_{R}$ has the same statistical definition under both hypotheses, whereas the target-scattering operator $G_{T}$ appears only under $H_{1}$:

(3) $$\begin{array}{cc} H_{0}:\;y_{B} & =G_{R}\left(\eta_{R}\right)x_{m}+c_{B}+w_{B},\\ H_{1}:\;y_{B} & =G_{R}\left(\eta_{R}\right)x_{m}+G_{T}\left(\eta_{T}\right)x_{m}+c_{B}+w_{B}. \end{array}$$

Here, $c_{B}$ contains target-independent clutter, residual multipath, and cancellation residuals, and

(4) $$w_{B}∼\mathrm{CN}\left(0,\Sigma_{B}\right).$$

A single-scattering-center target is represented by

(5) $$G_{T}\left(\eta_{T}\right)=\alpha_{T}D\left(\nu_{T}\right)T\left(\tau_{T}\right),\;\eta_{T}=\left(\tau_{T},\nu_{T},\alpha_{T}\right),$$

where $T(\tau_{T})$ and $D(\nu_{T})$ are fractional-delay and Doppler operators. The reference path may contain a line-of-sight component or sufficiently stable target-independent multipath or troposcatter. Components are assigned according to whether they retain the same statistical role under $H_{0}$ and $H_{1}$, not merely according to their geometric labels.

The detection model assumes one dominant scattering center and a complex gain that is constant over the 48-symbol pulse. Section 5.4 evaluates finite multi-center and Doppler-spread departures from this approximation; multi-target data association and extended-target inversion are outside the present receiver model.

Station A knows the transmitted message and observes

(6) $$y_{A}=\chi_{i}G_{A}\left(\eta_{A}\right)x_{m}+c_{A}+w_{A},\;\chi_{0}=0,\;\chi_{1}=1.$$

Because $G_{A}$ and $G_{T}$ describe different monostatic and bistatic geometries, Station A is not an upper bound on Station B. A known-message reference for Station B must be evaluated using the Station B geometry and covariance.

The whitened mean energies define the reference- and target-branch SNRs,

(7) $$\rho_{R}=E_{m}{∥G_{R}x_{m}∥}_{\Sigma_{B}^{-1}}^{2},\;\rho_{T}=E_{m}{∥G_{T}x_{m}∥}_{\Sigma_{B}^{-1}}^{2}.$$

These variables describe path availability continuously. Residual synchronization errors are included in the delay-Doppler search domain.

The observation partition can be implemented within a common receiver. The operators $Q_{R}$ and $Q_{T}$ may select disjointed delay-Doppler-angle regions from a common sampled data cube, or they may represent deterministic outputs of different matched processors. If their noise or residual-clutter terms overlap, the resulting statistics are correlated and must be modeled jointly. The background term is conditioned on training data or represented through the same covariance model under both target hypotheses; otherwise, a change in the assumed background could be mistaken for target evidence.

**Figure 1:**
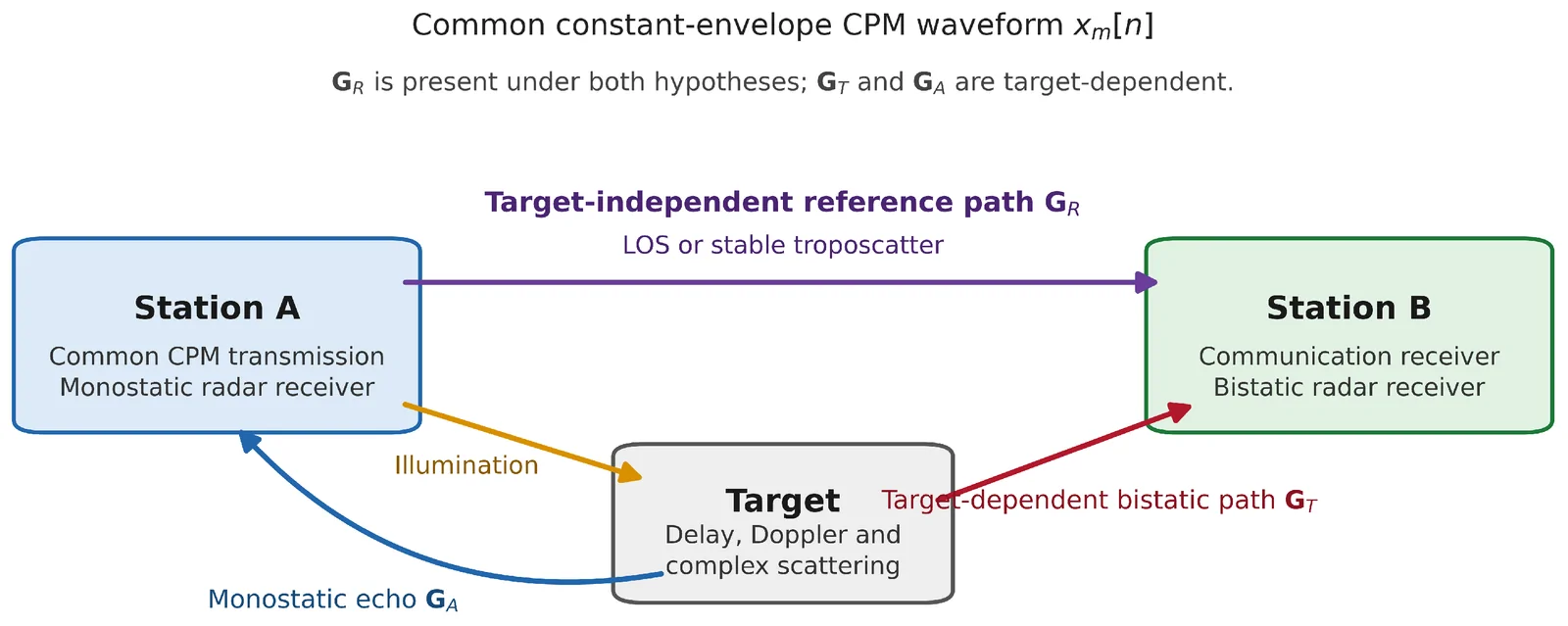
Target-independent reference and target-dependent scattering paths between Stations A and B, together with the monostatic path at Station A.

Figure 1 also identifies the four operating conditions covered by the model. A line-of-sight direct wave or stable target-independent propagation contributes to $G_{R}$. Target-scattered communication is represented by $G_{T}$ and is therefore available only under $H_{1}$. Monostatic and bistatic detection are described by $G_{A}$ and $G_{T}$, respectively. The same transmitted waveform and message model are used in every condition; only the path operators and available observations change.

### 2.2. Reference-Assisted Likelihood

Let

(8) $$y_{R}=Q_{R}y_{B},\;y_{T}=Q_{T}y_{B}$$

denote statistics associated with the reference and target search regions. Their outputs need not be independent. The posterior-assisted construction uses three conditions. First, the transmitted payload is generated independently of the target state, so

(9) $$p\left(m|H_{i},\eta_{i},\eta_{R}\right)=\pi_{m}.$$

Second, the reference statistic is target independent:

(10) $$p\left(y_{R}|m,H_{i},\eta_{i},\eta_{R}\right)=p_{R}\left(y_{R}|m,\eta_{R}\right),\;i\in \left\{0,1\right\}.$$

Third, a joint density for $y_{R}$ and $y_{T}$ exists on the parameter domain. These conditions concern the statistical role of the path and allow correlated reference and target statistics.

With message probability $\pi_{m}$, the reference posterior is

(11) $$\gamma_{m}\left(y_{R}\right)=\frac{\pi_{m}p_{R}\left(y_{R}|m,\eta_{R}\right)}{\sum_{a\in M}\pi_{a}p_{R}\left(y_{R}|a,\eta_{R}\right)}.$$

For random uncoded payloads without source side information, $\pi_{m}$ is uniform over the valid message set. This prior specifies only the ensemble over which the unknown message is marginalized.

**Proposition 1.**  *If the message is independent of the target hypothesis and parameters, the conditional reference density $p_{R}\left(y_{R}|m,\eta_{R}\right)$ is target-hypothesis invariant, and the joint density exists, then*

(12) $$p\left(y_{R},y_{T}|H_{i},\eta_{i}\right)=p\left(y_{R}\right)\sum_{m}\gamma_{m}\left(y_{R}\right)p\left(y_{T}|y_{R},m,H_{i},\eta_{i}\right).$$

The result follows by expanding the joint density over *m* and applying Bayes’ rule. The common factor $p(y_{R})$ cancels in the likelihood ratio, giving

(13) $$\Lambda_{B}^{R}=\frac{\max_{\eta_{1}}\sum_{m}\gamma_{m}\left(y_{R}\right)p\left(y_{T}|y_{R},m,H_{1},\eta_{1}\right)}{\max_{\eta_{0}}\sum_{m}\gamma_{m}\left(y_{R}\right)p\left(y_{T}|y_{R},m,H_{0},\eta_{0}\right)}.$$

Conditional independence permits replacement of the last density by $p_{T}\left(y_{T}|m,H_{i},\eta_{i}\right)$. When the statistics are correlated, the conditional Gaussian mean and covariance must be used; multiplying independent reference and target terms after both were formed from shared samples would count part of the observation twice.

For jointly complex-Gaussian statistics with block covariance

(14) $$\Sigma =\left(\begin{array}{cc} \Sigma_{RR} & \Sigma_{RT}\\ \Sigma_{TR} & \Sigma_{TT} \end{array}\right),$$

the target statistic conditioned on the reference has

(15) $$\mu_{T|R,i}=\mu_{T,i}+\Sigma_{TR}\Sigma_{RR}^{-1}\left(y_{R}-\mu_{R}\right),$$

(16) $$\Sigma_{T|R}=\Sigma_{TT}-\Sigma_{TR}\Sigma_{RR}^{-1}\Sigma_{RT}.$$

This conditional form accounts once for the information shared by the two statistics. The independent-branch simplification follows only when the cross covariance and any shared random component vanish.

### 2.3. Joint Hypotheses Without a Reference

When $\rho_{R}=0$, the only message-bearing observation at Station B is the target-scattered component under $H_{1}$:

(17) $$\begin{array}{cc} H_{0}:\; & y_{T}=c_{B}+w_{B},\\ H_{1}\left(m,\eta_{T}\right):\; & y_{T}=\alpha_{T}D\left(\nu_{T}\right)T\left(\tau_{T}\right)x_{m}+c_{B}+w_{B}. \end{array}$$

The appropriate generalized likelihood ratio marginalizes the random message and profiles the deterministic target parameters,

(18) $$\Lambda_{B}^{NR}\left(y_{T}\right)=\frac{\max_{\eta_{T}\in \Omega_{T}}\sum_{m\in M}\pi_{m}p_{1}\left(y_{T}|m,\eta_{T}\right)}{p_{0}\left(y_{T}\right)}.$$

After a target-present decision, the joint estimate is

(19) $$\left(\hat{m},{\hat{\eta }}_{T}\right)={\arg\max}_{m,\eta_{T}}\left\{\log\pi_{m}+\log p_{1}\left(y_{T}|m,\eta_{T}\right)\right\}.$$

Under $H_{0}$, $y_{T}$ is independent of *m*; under $H_{1}$, its message posterior depends on the target-scattered template. Except in the degenerate case in which all message-conditioned densities are identical, no target-hypothesis-invariant posterior can be derived from $y_{T}$ and reused as an independent input to a second likelihood evaluation. The joint marginal likelihood therefore replaces decode-then-detect factorization when no target-independent reference exists.

To see why a sequential reuse of $y_{T}$ fails, note that

(20) $$p\left(m|y_{T},H_{0}\right)=\pi_{m},$$

whereas

(21) $$p\left(m|y_{T},H_{1},\eta_{T}\right)=\frac{\pi_{m}p_{1}\left(y_{T}|m,\eta_{T}\right)}{\sum_{a}\pi_{a}p_{1}\left(y_{T}|a,\eta_{T}\right)}.$$

The latter varies with both the observation and the message whenever the scattered templates are distinguishable. Hence, the two hypotheses have no common message posterior derived from $y_{T}$. The finite-state implementation in Section 4 evaluates the joint marginal likelihood directly and uses the target-scattered samples once.

Performance is reported through $P_{D}$, $P_{FA}$, target-parameter RMSE, conditional message BLER, and

(22) $$P_{\mathrm{joint}}=\mathrm{Pr}\left(\hat{H}=H_{1},\hat{m}=m,d_{\eta }\left({\hat{\eta }}_{T},\eta_{T}\right)\leq \epsilon_{\eta }|H_{1}\right).$$

The message-conditioned known-parameter Neyman–Pearson result and coherent communication formulas serve as same-geometry reference curves for interpreting the full joint receiver.

### 2.4. Statistical Effect of Message Uncertainty

For equal-covariance Gaussian simple hypotheses and a specified message, the detection separation is the whitened mean-difference energy

(23) $$D_{01}\left(m\right)={∥\mu_{1,m}-\mu_{0,m}∥}_{\Sigma^{-1}}^{2}.$$

When the message is unknown, the target-present density is a mixture. Its KL divergence from the target-absent density satisfies

(24) $$D_{det}=\sum_{m}w_{m}D_{KL}\left(p_{1}\left(\cdot |m\right)∥p_{0}\right)-I\left(M;Y|I,H_{1}\right).$$

Let $\pi_{m}=\mathrm{Pr}\left(M=m|I\right)$ denote the message probability obtained from the reference statistic I, and let

(25) $$p_{B}=\sum_{m}\pi_{m}p_{m}$$

denote the resulting target-present mixture. The separation lost by marginalizing the message is the conditional mutual information between the message and the target observation under the target-present hypothesis and is nonnegative. Direct expansion gives

(26) $$\sum_{m}\pi_{m}D\left(p_{m}∥p_{0}\right)-D\left(p_{B}∥p_{0}\right)=\sum_{m}\pi_{m}D\left(p_{m}∥p_{B}\right)=I\left(M;Y|I,H_{1}\right)\geq 0.$$

For a continuous target parameter, let $s_{\mathrm{complete}}$ denote the score when the message is known. The mixture score is the posterior mean of $s_{\mathrm{complete}}$. Consequently,

(27) $$J_{\mathrm{mix}}=E\left[J_{\mathrm{complete}}\right]-E\left[\mathrm{Cov}\left(s_{\mathrm{complete}}|Y,I\right)\right],$$

where $J_{\mathrm{mix}}$ and $J_{\mathrm{complete}}$ are the Fisher-information matrices for the unknown-message and known-message models, respectively. The covariance term is positive semidefinite, so message marginalization cannot increase Fisher information. This identity motivates posterior-assisted processing; detection probability and estimation RMSE remain the operational measures.

## 3. Constant-Envelope CPM Waveform Design

### 3.1. Finite-State Pulsed CPM

Let $T_{c}$ be the CPM input-symbol interval and

(28) $$a_{k}\left(m\right)\in A_{M}=\left\{\pm 1,\pm 3,\ldots ,\pm \left(M-1\right)\right\}.$$

The rational modulation index is h=r/p, and the phase response

(29) $$q\left(t\right)=\int_{-\infty }^{t}g\left(\lambda \right)d\lambda$$

is generated by a finite-support frequency pulse satisfying

(30) $$\int g\left(t\right)dt=\frac{1}{2}.$$

For the selected length-two raised-cosine frequency pulse,

(31) $$g_{2RC}\left(t\right)=\frac{1}{4T_{c}}\left[1-\cos\left(\frac{\pi t}{T_{c}}\right)\right],\;0\leq t\leq 2T_{c},$$

and $g_{2RC}\left(t\right)=0$ elsewhere. Its integral is 1/2, ensuring the conventional CPM phase normalization.

The total active-pulse phase is

(32) $$\Phi_{m}\left(t\right)=\phi_{r}\left(t;c\right)+2\pi h\sum_{k}a_{k}\left(m\right)q\left(t-kT_{c}\right)+\phi_{0},$$

where $\phi_{r}\left(t;c\right)$ is a deterministic radar phase shared by every message. The energy-normalized waveform is

(33) $$x_{m}^{\left(E\right)}\left(t\right)=\sqrt{\frac{E}{T_{p}}}e^{j\Phi_{m}\left(t\right)}1_{\left[0,T_{p}\right)}\left(t\right).$$

Because the common radar phase multiplies every branch template by a known unit-modulus factor, it changes the delay-Doppler waveform while preserving the CPM state connectivity. At symbol time *k*, the state

(34) $$s_{k}=\left(\vartheta_{k},a_{k-L_{c}+1},\ldots ,a_{k-1}\right),\;\vartheta_{k}=\pi h\sum_{i\leq k-L_{c}}a_{i}\;\mathrm{mod}\;2\pi$$

contains the accumulated phase and the CPM memory symbols. The number of possible states is at most 16 for the adopted parameters, with the reachable set determined by the initial state and branch recursion [40,41,42].

The evaluated waveform uses M=4, h=1/2, $L_{c}=2$, and a length-two raised-cosine frequency pulse. Each pulse contains $K_{\mathrm{info}}=46$ information-bearing input symbols and $K_{\mathrm{tail}}=2$ known termination symbols, yielding

(35) $$B_{p}=K_{\mathrm{info}}\log_{2}M=92\;\mathrm{bits}$$

over 48 transmitted symbols. The termination symbols return the trellis to a known state and permit independent pulse resets. For $T_{p}=40\;\mu s$ and duty cycle D=0.1, the pulse repetition frequency is 2.5 kHz and a pulsewise updated traffic stream carries 230 kbit/s before frame-level overhead.

Let $N_{\mathrm{pay}}$ of $N_{\mathrm{frm}}$ pulses carry independently updated traffic. The useful traffic rate and active-pulse spectral efficiency are

(36) $$R_{\mathrm{frm}}=\frac{N_{\mathrm{pay}}}{N_{\mathrm{frm}}}\frac{DB_{p}}{T_{p}},\;\eta_{\mathrm{act}}=\frac{B_{p}}{T_{p}{\bar{B}}_{99}},$$

where ${\bar{B}}_{99}$ is the message-ensemble-average 99% occupied bandwidth. These definitions account explicitly for CPM termination, duty cycle, and pulse scheduling. Repeating one message across a coherent processing interval improves weak scattered-link reliability but reduces the new-information rate and is evaluated separately from pulsewise updated traffic.

### 3.2. Random-Message Risk and Feasible Waveform Set

The design variable is θ=(h,β,c), where *h* is selected from a finite rational set, β controls the finite-support frequency pulse, and *c* parameterizes the common radar phase. In the evaluated two-parameter phase family,

(37) $$\phi_{r}\left[n;c\right]=c_{1}\pi u_{n}^{2}+c_{2}\sin\left(2\pi u_{n}\right),\;u_{n}=\frac{n}{N-1}.$$

All messages share the same θ, and

(38) $$x_{m}\left[n;\theta \right]=\exp\left[j\phi_{r}\left[n;c\right]+j2\pi h\sum_{k}a_{k}\left(m\right)q\left(nT_{s}-kT_{c};\beta \right)+j\phi_{0}\right]$$

satisfies $|x_{m}\left[n;\theta \right]|=1$ identically.

For delay index *l* and Doppler $f_{d}$, the sampled ambiguity function is

(39) $$\chi_{m}\left(l,f_{d};\theta \right)=\sum_{n\in I_{l}}x_{m}\left[n;\theta \right]x_{m}^{*}\left[n-l;\theta \right]e^{-j2\pi f_{d}nT_{s}},$$

where $I_{l}$ is the valid overlap set. A guard region excludes the mainlobe from the optimization set, ensuring that the objective measures sidelobe energy without favoring mainlobe broadening.

For message *m*, define the normalized ambiguity-sidelobe loss over a prescribed delay-Doppler set $S_{sl}$:

(40) $$L_{rad}\left(m;\theta \right)=\sum_{\left(l,f_{d}\right)\in S_{sl}}w_{l,f}\frac{|\chi_{m}\left(l,f_{d};\theta \right){|}^{2}}{|\chi_{m}{\left(0,0;\theta \right)|}^{2}},\;\sum w_{l,f}=1.$$

The random payload makes the ambiguity-sidelobe loss message dependent. On a fixed training set, its empirical upper-tail risk is defined using CVaR [43].

(41) $${\hat{\mathrm{CVaR}}}^{\alpha }\left(\theta \right)=\min_{t}\left[t+\frac{1}{\left(1-\alpha \right)N_{tr}}\sum_{m\in M_{tr}}{\left(L_{rad}\left(m;\theta \right)-t\right)}_{+}\right],$$

with α=0.8. CVaR is used here as a stochastic design functional for high-loss messages; PSL, ISL, mainlobe width, $P_{D}$, and RMSE remain separate evaluation metrics. The objective is

(42) $$J^{rad}\left(\theta \right)=\frac{1}{N_{tr}}\sum_{m}L_{rad}\left(m;\theta \right)+\lambda_{tail}{\hat{\mathrm{CVaR}}}^{0.8}\left(\theta \right).$$

The mean term controls the message-ensemble average, while the CVaR term penalizes the upper 20% probability mass without assigning a different waveform to each message. Training and validation messages are disjointed. Separate training and validation sets are required because evaluation on the optimization messages can overstate a small tail-risk change.

Communication and spectral feasibility are imposed through the bitwise GMI of the finite-state receiver and the mean out-of-band energy fraction,

(43) $$\begin{array}{cc} \min_{\theta }\; & J^{rad}\left(\theta \right)\\ s.t.\;\; & R_{\mathrm{GMI}}^{tr}\left(\theta ;4\;\mathrm{dB}\right)\geq R_{0},\\ \; & E_{\mathrm{OOB}}^{tr}\left(\theta \right)\leq \epsilon_{\mathrm{OOB}},\\ \; & h\in H,\;0\leq \beta \leq \beta_{\max},\;c\in C. \end{array}$$

If $B_{j}$ is a mapped bit and $L_{j}$ is the finite-state receiver log-likelihood ratio, the empirical bitwise GMI is

(44) $$R_{\mathrm{GMI}}=\sum_{j=1}^{\log_{2}M}\left[1-\frac{1}{N_{ch}}\sum_{r=1}^{N_{ch}}\log_{2}\left(1+e^{-\left(1-2B_{j,r}\right)L_{j,r}}\right)\right].$$

The spectral quantity is

(45) $$E_{\mathrm{OOB}}=\frac{\frac{1}{N_{tr}}\sum_{m}\int_{f\notin B_{0}}{\left|X_{m}\left(f\right)\right|}^{2}df}{\int_{-\infty }^{\infty }{\left|X_{m}\left(f\right)\right|}^{2}df}.$$

GMI and out-of-band energy define feasibility; neither is substituted for joint target-message performance.

The thresholds are fixed before optimization as 99% of the standard-CPM training GMI and 105% of its training out-of-band energy. Training uses 256 fixed messages for radar risk and spectrum and 96 independent frames for GMI. Validation uses 2048 new messages and 256 new communication frames.

The low-dimensional nonconvex problem is solved by hierarchical enumeration of the structural parameters, a complete coarse grid over the common-phase domain, and local grid refinement around the best feasible point. Each candidate uses the same training samples. The selected point,

(46) $$\left(\hat{h},\hat{\beta },{\hat{c}}_{1},{\hat{c}}_{2}\right)=\left(0.5,0,0,6.25\right),$$

is the best feasible candidate within the specified domain and grid; no claim of continuous-domain global optimality is made. The adjacent point with lower training risk violates the spectral constraint, making that constraint active at the adopted grid spacing.

### 3.3. Hierarchical Numerical Solution

The structural parameters are first enumerated on their prescribed finite sets. For each structure, the complete common-phase domain is searched on a coarse grid, followed by a local refinement around the best feasible point. Every candidate is evaluated on the same training messages and channel samples, and feasibility is checked before risk ranking. If $N_{tr}$ messages, $N_{sl}$ sidelobe samples, $N_{\mathrm{GMI}}$ communication frames, and $N_{cand}$ parameter candidates are used, the leading cost is

(47) $$O\left(N_{cand}N_{tr}N_{sl}N+N_{\mathrm{GMI}}K\left|S\right|MN_{s}+N_{tr}N_{FFT}\log N_{FFT}\right).$$

This offline search is tractable because the phase family is low dimensional. Independent validation is performed once at the selected point, without changing the thresholds or grid center.

### 3.4. Directed Conditional Event Distance

Waveform risk and receiver message errors describe different effects. To analyze the latter, consider a true joint alternative ζ=(m,η) and a competing alternative $\zeta^{\prime }=\left(m^{\prime },\eta^{\prime }\right)$ under $H_{1}$. After whitening, define the directed distance

(48) $$D_{J}\left(\zeta ∥\zeta^{\prime }\right)=∥\mu_{R}\left(\zeta \right)-\mu_{R}\left(\zeta^{\prime }\right){∥}^{2}+{∥\mu_{T}\left(\zeta \right)-\mu_{T}\left(\zeta^{\prime }\right)∥}^{2},$$

for conditionally independent reference and target branches. With branch energies represented by $\rho_{R}$ and $\rho_{T}$,

(49) $$D_{J}\left(\zeta ∥\zeta^{\prime }\right)=\rho_{R}D_{R}\left(m,m^{\prime }\right)+\rho_{T}D_{T}\left(\zeta ,\zeta^{\prime }\right).$$

Consequently, the distance reduces continuously to the target-branch term as $\rho_{R}\to 0$, whereas a reference observation that distinguishes the two paths contributes increasingly with $\rho_{R}$.

For two fixed Gaussian alternatives with common covariance, the log-likelihood-ratio error under the true alternative has pairwise probability

(50) $$Q\left(\sqrt{\frac{D_{J}}{2}}\right).$$

In the receiver, however, each competing message path is accompanied by unknown target parameters. The relevant local diagnostic is therefore

(51) $$d_{dir}^{2}\left(\zeta ∥\zeta^{\prime }\right)=\min_{\eta^{\prime }\in \Omega^{\prime }}{∥\mu \left(\zeta \right)-\mu \left(\zeta^{\prime },\eta^{\prime }\right)∥}_{\Sigma^{-1}}^{2},$$

which evaluates how closely a competing path can match data generated by the specified true event. The minimization makes the measure asymmetric in general.

The competing delay, Doppler, and complex gain are profiled for every competing event. For true template $u_{e}$ and competing template $v_{e}\left(\tau^{\prime },\nu^{\prime }\right)$, analytical elimination of the competing gain gives

(52) $$d_{e}^{2}=\min_{\tau^{\prime },\nu^{\prime }}\left[{∥u_{e}∥}^{2}-\frac{|v_{e}^{H}u_{e}{|}^{2}}{{∥v_{e}∥}^{2}}\right].$$

This projection residual is nonnegative and vanishes only when the two templates occupy the same one-dimensional complex subspace. With a fixed known common gain and no target-parameter search, it reduces to the classical CPM Euclidean event distance. The metric is directed because the nuisance parameters are profiled for the competing event conditional on a specified true event. It is used to rank finite competing events and to interpret receiver behavior, not as a global BLER formula.

For a nonzero competing template, expand the squared residual as a quadratic function of the competing complex gain. Setting the Wirtinger derivative with respect to the conjugate gain to zero gives the least-squares projection coefficient. Substitution yields the orthogonal-projection residual in the preceding equation, which is nonnegative by the Cauchy–Schwarz inequality and vanishes only for collinear whitened templates. With fixed gain and delay-Doppler parameters, the projection step disappears and the expression reduces to the conventional CPM Euclidean event distance. The local interpretation requires a common covariance, an identifiable nearest competing point, and the stated parameter-search domain.

## 4. Joint Target-Message Receiver

### 4.1. Message-Conditioned Processing at Station A

Station A reconstructs the complete transmitted vector $x_{m}$. For candidate delay and Doppler,

(53) $$v_{A}\left(m,\tau ,\nu \right)=D_{A}\left(\nu \right)T_{A}\left(\tau \right)G_{A}x_{m}.$$

With unknown complex target gain and covariance $\Sigma_{A}$, profiling the gain yields

(54) $$T_{A}\left(m,\tau ,\nu \right)=\frac{|v_{A}^{H}\Sigma_{A}^{-1}\left(y_{A}-c_{A}\right){|}^{2}}{v_{A}^{H}\Sigma_{A}^{-1}v_{A}},\;T_{A}^{⋆}\left(m\right)=\max_{\tau ,\nu }T_{A}\left(m,\tau ,\nu \right).$$

Message conditioning removes template uncertainty but not the message dependence of the ambiguity function or sidelobes. Station A and Station B are therefore evaluated under their respective geometries.

The complete CPM phase, including the communication symbols and common radar phase, is retained in the matched template. Thus, knowledge of the message removes message-template mismatch while retaining the complete communication modulation in the echo template. The target threshold is calibrated under Station A’s own background and geometry.

### 4.2. Unified Finite-State Recursion at Station B

Let $b_{k}=\left(s_{k-1},s_{k}\right)$ denote a valid CPM branch and $x_{k}\left(b_{k}\right)$ its sample template. For an available reference path, the branch log metric is

(55) $$\lambda_{R,k}\left(b_{k}\right)=\log P\left(b_{k}|s_{k-1}\right)+\log p_{R}\left(y_{R,k}|b_{k},\eta_{R}\right).$$

The reference forward and backward metrics are

(56) $$A_{R,k}\left(s\right)={\mathrm{LSE}}_{b_{k}:s_{k}=s}\left[A_{R,k-1}\left(s_{k-1}\right)+\lambda_{R,k}\left(b_{k}\right)\right],$$

(57) $$B_{R,k-1}\left(s^{\prime }\right)={\mathrm{LSE}}_{b_{k}:s_{k-1}=s^{\prime }}\left[\lambda_{R,k}\left(b_{k}\right)+B_{R,k}\left(s_{k}\right)\right].$$

They produce branch posteriors and bit LLRs for communication reporting. In the exact target likelihood, the reference branch metrics and state connectivity are retained directly; independent bit LLRs are not multiplied as if the CPM memory had disappeared.

For a fixed target-parameter candidate ζ=(τ,ν,α), the joint branch metric is

(58) $$\lambda_{i,k}^{R}\left(b_{k};\zeta_{i}\right)=\lambda_{R,k}\left(b_{k}\right)+\log p\left(y_{T,k}|y_{R},b_{k},H_{i},\zeta_{i}\right).$$

Without a reference,

(59) $$\lambda_{1,k}^{NR}\left(b_{k};\zeta \right)=\log P\left(b_{k}|s_{k-1}\right)+\log p_{T}\left(y_{T,k}|b_{k},H_{1},\zeta \right),$$

while $\ell_{0}^{NR}=\log p_{0}\left(y_{T}\right)$ is evaluated directly. At each parameter point, the log-domain forward recursion is

(60) $$A_{i,k}^{r}\left(s;\zeta_{i}\right)={\mathrm{LSE}}_{b_{k}:s_{k}=s}\left[A_{i,k-1}^{r}\left(s_{k-1};\zeta_{i}\right)+\lambda_{i,k}^{r}\left(b_{k};\zeta_{i}\right)\right].$$

With prescribed initial and terminal states, $A_{i,K}^{r}\left(s_{f};\zeta_{i}\right)$ equals the message-marginal log-likelihood over all valid paths. The complex gain is shared over the frame, so the recursion is completed at fixed α before the outer maximization:

(61) $$\ell_{i}^{r,⋆}=\max_{\zeta_{i}\in G_{i}}A_{i,K}^{r}\left(s_{f};\zeta_{i}\right),\;\Lambda_{B}^{r}=\ell_{1}^{r,⋆}-\ell_{0}^{r,⋆}.$$

The threshold is calibrated from independent $H_{0}$ samples at the specified false-alarm probability. After an $H_{1}$ decision, a backward recursion at $\hat{\zeta }$ produces branch and bit posteriors, and maximum-path traceback produces $\hat{m}$. As $\rho_{R}$ increases, the reference posterior concentrates and the recursion approaches the same-geometry known-message receiver. Removing the reference metric gives the joint no-reference likelihood in Section 2.

Table 1 summarizes the finite-state receiver at Station B. The same recursion uses reference-derived branch metrics when the target-independent path is available and the message prior when it is absent. Direct enumeration is used only to verify the message marginal on the short unit frame.

For *K* symbols, |S| states, *M* outgoing branches, and an $N_{\tau }N_{\nu }N_{\alpha }$ target grid, the time complexity is

(62) $$O(N_{\tau }N_{\nu }N_{\alpha }K|S|M).$$

Rolling forward metrics require O(|S|) state memory, while storing full-frame backward metrics and traceback information requires O(K|S|). For *R* conditionally independent receiving branches, branch-metric evaluation grows approximately linearly with *R*, whereas the CPM state count is unchanged. Correlated branches are represented through the joint covariance used in the likelihood.

In the strong-reference limit, Bayes’ rule makes the reference posterior concentrate on the transmitted path, and the joint recursion approaches the known-message Station B receiver. At finite reference SNR, both branches contribute to path discrimination. Setting the reference metric to zero yields the no-reference likelihood without changing the target-branch state graph. These limits form one continuous receiver family indexed by reference-path strength.

A state-mass approximation retains the smallest state set whose normalized forward mass is at least 1−ϵ. Because the complete trellis has only 16 states, pruning is evaluated for likelihood fidelity and end-to-end agreement and is not presumed to reduce runtime. The one-shot max-log receiver retains a single message path before target processing and therefore provides the principal comparison for the effect of preserving message uncertainty.

The pruning approximation uses the normalized forward mass

(63) $$\omega_{k}\left(s\right)=\frac{e^{A_{k}\left(s\right)}}{\sum_{s^{\prime }}e^{A_{k}\left(s^{\prime }\right)}}$$

and retains the smallest state set whose cumulative mass is at least 1−ϵ. Because future samples can reorder partial paths, the retained mass at one step is not treated as a full-frame error bound; likelihood differences and end-to-end decisions are checked against the complete recursion.

The one-shot baseline selects one message path, reconstructs its target template, and evaluates

(64) $$T_{HD}=\max_{\tau ,\nu }\frac{|{\hat{v}}^{H}\Sigma^{-1}\left(y_{T}-c_{T}\right){|}^{2}}{{\hat{v}}^{H}\Sigma^{-1}\hat{v}}.$$

It uses the same observations and parameter grid as the joint receiver but discards all competing message paths before the target test. The comparison in Section 5 therefore isolates the effect of preserving message uncertainty.

## 5. Results

### 5.1. Evaluation Conditions

Table 2 summarizes the waveform, full-frame receiver, statistical, and system-level evaluation conditions. All pulses have 40μs duration and 10% duty cycle, and radar processing uses 32 pulses. The CPM frame contains 46 information symbols and two termination symbols, carrying 92 information bits. The six-bit frame is used only for direct-enumeration verification; the full-frame receiver performance curves use the complete 92-bit frame.

For every full-frame receiver and reference condition, the threshold is the higher empirical quantile from 1000 target-absent calibration frames. False alarms are evaluated on a separate 1000-frame bank, and each target-SNR point contains 300 target-present frames. Probability intervals use the Wilson method; receiver differences use 10,000 paired frame-level bootstrap resamples, and RMSE intervals use 4000 frame-level resamples among detections. Calibration and evaluation samples are independent.

Table 2 states the waveform-specific comparison controls. Standard CPM uses the same alphabet, state memory, and frame structure as the proposed waveform, isolating the effect of common-phase selection.

### 5.2. Waveform Design and Basic Characteristics

The proposed and standard CPM waveforms are both strictly constant envelope and continuous phase; their intrapulse PAPR and envelope variation are numerically indistinguishable from zero. These common CPM properties verify the construction; the effect of the optimized common phase is evaluated through independent-message statistics. The proposed waveform has a 99% occupied bandwidth of 2.65 MHz for the representative message, compared with 2.50 MHz for standard CPM and 2.64 MHz for the bandwidth-matched LFM waveform.

**Figure 2:**
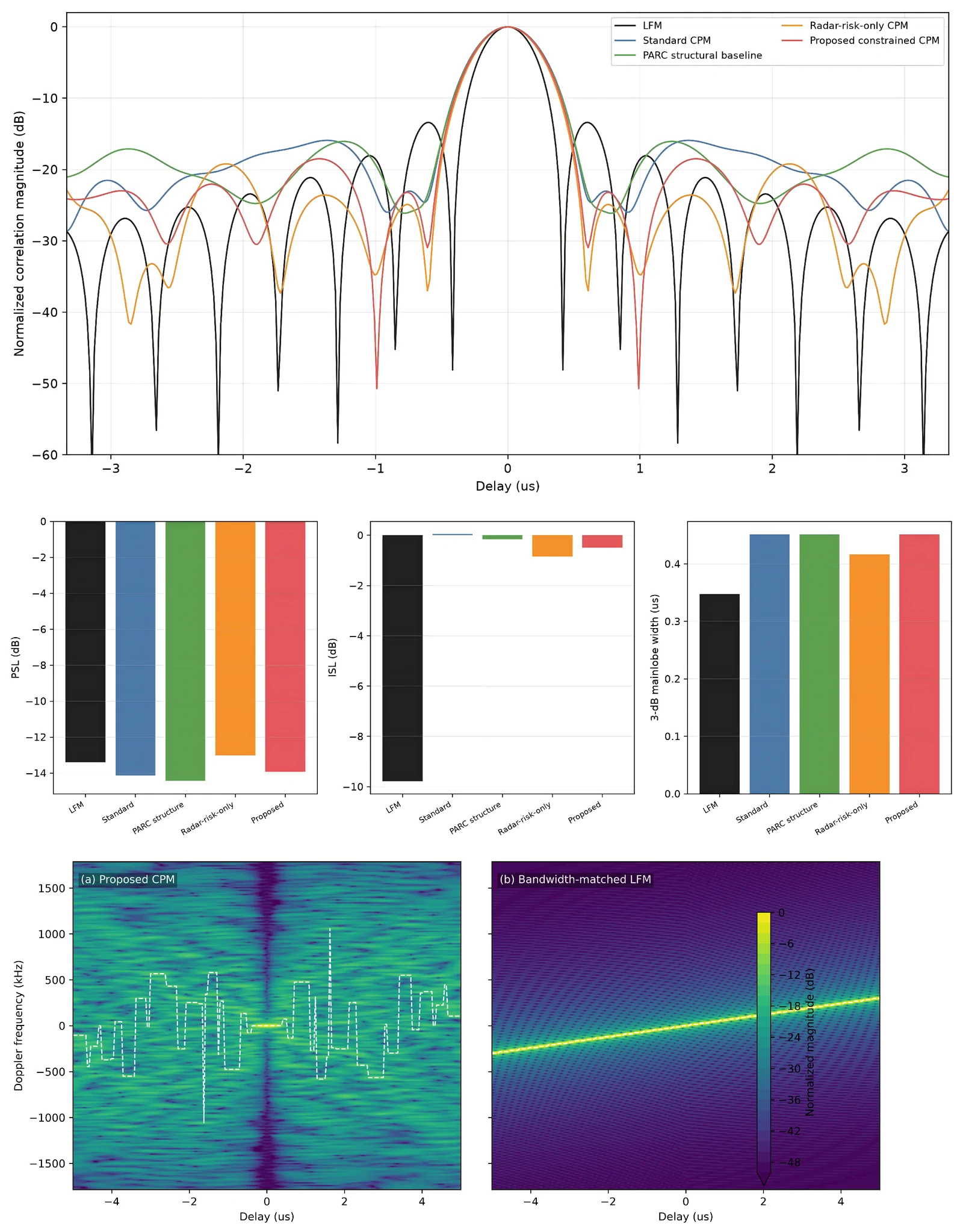
Radar waveform characteristics showing zero-Doppler autocorrelation, mainlobe and sidelobe metrics, and two-dimensional ambiguity functions of the proposed CPM and bandwidth-matched LFM waveforms.

Figure 2 compares zero-Doppler autocorrelation, conventional radar metrics, and two-dimensional ambiguity functions. The proposed CPM has PSL −13.94 dB and ISL −0.50 dB; standard CPM has −14.14 dB and 0.06 dB, and LFM has −13.40 dB and −9.79 dB. The proposed waveform therefore does not uniformly dominate either reference: its ISL is 0.56 dB below standard CPM but 9.29 dB above LFM. The rectangular LFM result exhibits the expected oblique delay-Doppler ridge,

(65) $$|\chi_{L}\left(\tau ,\nu \right)|=\frac{T_{p}-\left|\tau \right|}{T_{p}}\mathrm{sinc}\left[\left(\nu -\mu \tau \right)(T_{p}-|\tau |)\right],\;\left|\tau \right|<T_{p},$$

whereas CPM produces a message-dependent multipeak surface. On the complete 241-by-241 grid, numerical LFM integration agrees with the analytical magnitude with a normalized RMSE of $1.63\times 10^{-4}$ and a maximum absolute error of $9.41\times 10^{-4}$; the numerical and analytical ridge slopes differ by 0.10%. Display decimation is excluded from these calculations. This check validates the LFM comparison baseline and is not evidence for the proposed waveform.

**Figure 3:**
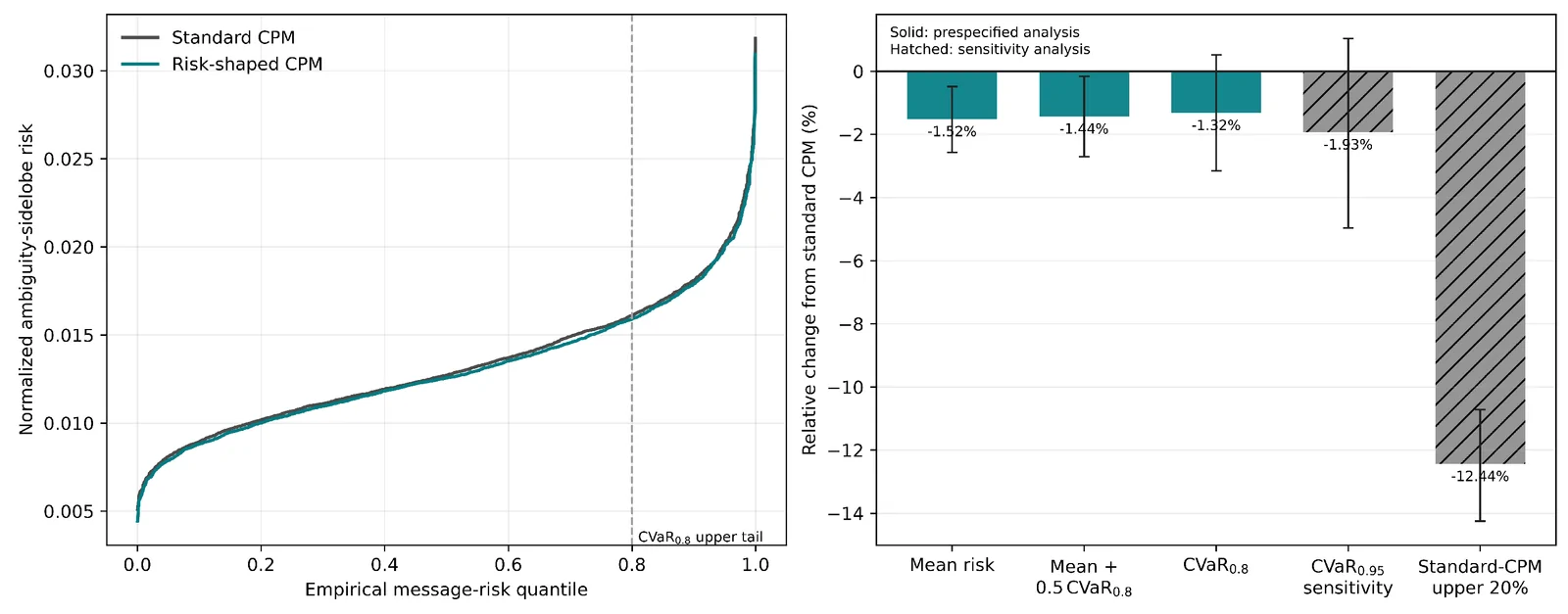
Independent-message risk quantiles and paired changes in the random-message objective relative to standard CPM.

Figure 3 gives the evidence specific to common-phase risk shaping. On 2048 independent messages, the prescribed mean-${\mathrm{CVaR}}_{0.8}$ objective decreases from $2.27\times 10^{-2}$ to $2.24\times 10^{-2}$, a relative change of −1.44% with a paired 95% bootstrap interval of [−2.70%,−0.16%]. Mean risk decreases by 1.52%, whereas the 1.32% reduction in the CVaR term alone has an interval that includes zero. Risk decreases for 52.83% of individual messages, and the mean over the subset fixed in advance as the highest-risk 20% under standard CPM decreases by 12.44%. The optimization thus redistributes message-dependent sidelobe risk.

The proposed waveform retains the standard-CPM validation GMI of 1.82 bit/transmitted symbol at 4 dB. Its mean out-of-band energy increases by 4.90%, remaining within the prescribed 5% tolerance. A candidate selected without the spectral constraint reduces training risk further but increases out-of-band energy by 11.04%; thus, the final point is determined by the active spectral constraint.

**Figure 4:**
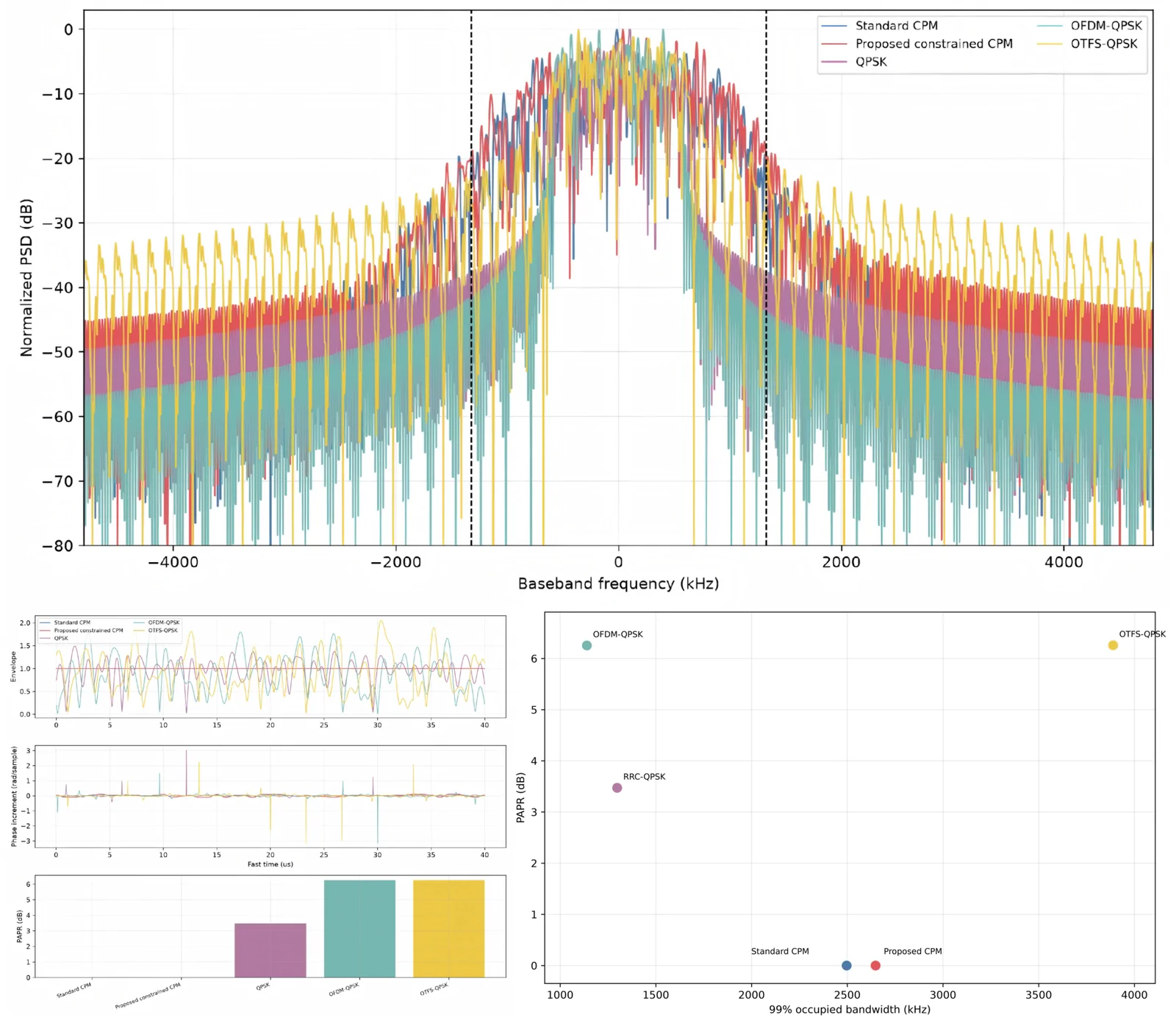
Communication-waveform comparison at equal pulse duration and net payload: envelope and phase behavior, spectrum, PAPR, and occupied bandwidth.

At equal 40μs duration and 92-bit payload, the proposed CPM has a 99% occupied bandwidth of 2.65 MHz, compared with 1.30 MHz for RRC-QPSK; its net active-pulse spectral efficiency is therefore approximately 49% of the RRC-QPSK value. OFDM and OTFS provide flexible multicarrier processing but have higher finite-frame PAPR in the evaluated configuration. Figure 4 separates these transmitter and bandwidth costs from receiver performance. The corresponding costs of constant-envelope CPM are finite-state memory, termination overhead, and greater occupied bandwidth than pulse-shaped QPSK.

### 5.3. Communication, Detection, and Joint Recovery

The finite-state implementation was first checked against direct enumeration of all 64 valid messages on the six-bit unit frame. Across 32 trials, the maximum target-present and target-absent log-likelihood differences are below $9\times 10^{-16}$, the maximum branch-posterior error is $1.3\times 10^{-15}$, and every maximum-posterior message agrees. These checks establish implementation correctness but are not used as performance results. Table 3 reports the complete 92-bit recursion and state-mass approximations at one fixed operating point.

The complete recursion is faster than the tested pruning implementation because sorting overhead exceeds the saving from discarding states on this small trellis. State-mass pruning is therefore retained only as a validated approximation. The $\epsilon =10^{-4}$ setting preserves the tested decisions with sub-0.002 likelihood error, whereas $\epsilon =10^{-2}$ changes BLER by 0.075.

**Figure 5:**
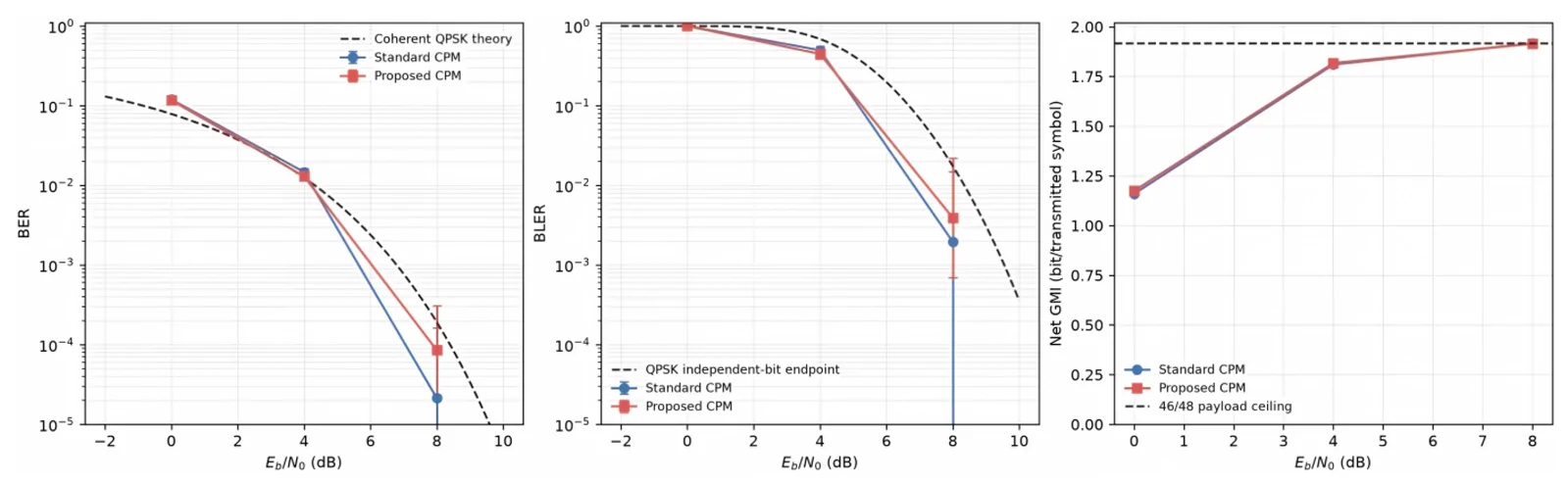
Reference-path BER, BLER, and net GMI for the 92-bit CPM frame, with a coherent-QPSK reference.

Figure 5 reports reference-path BER, BLER, and net GMI for the 46 + 2-symbol frame. The proposed and standard CPM curves are effectively coincident, indicating unchanged reference-link communication performance at the evaluated operating points. The coherent-QPSK curve is a modulation reference rather than a bound for the finite-state CPM receiver.

**Figure 6:**
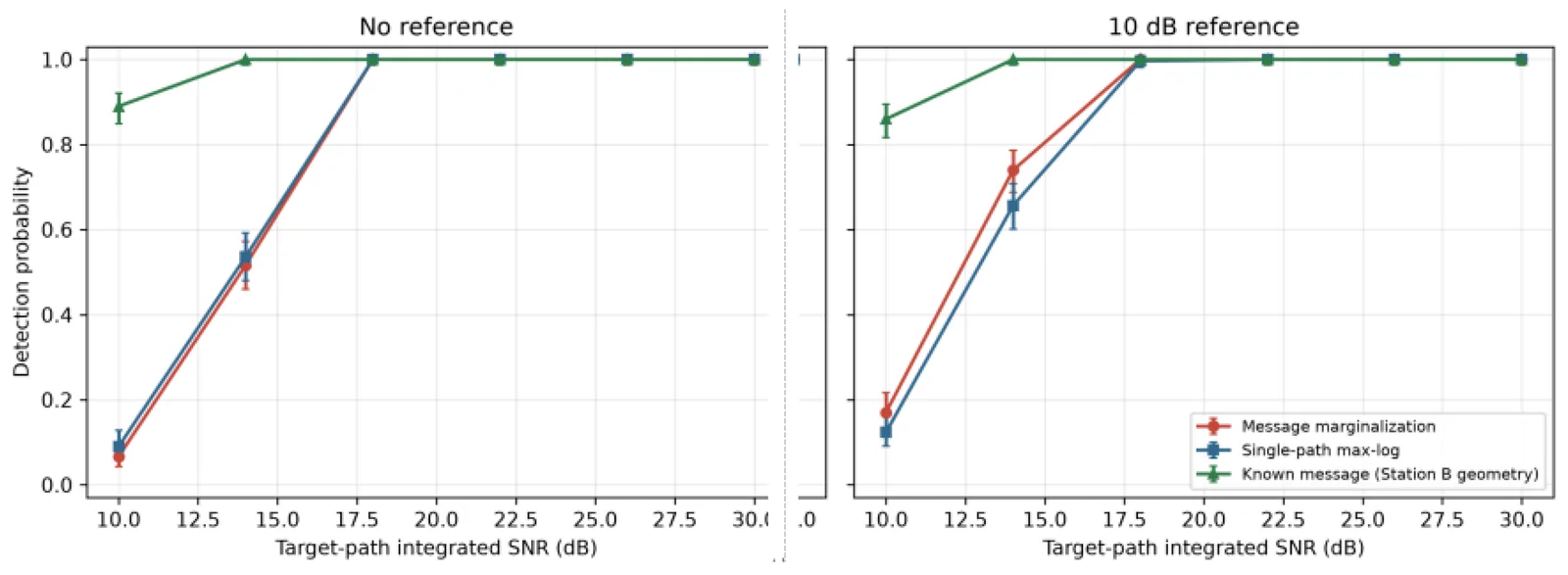
Full-frame detection probability at $P_{FA}=0.01$ for no-reference and 10 dB reference-path cases. Each point uses 300 independent 92-bit $H_{1}$ frames; error bars are 95% Wilson intervals.

**Figure 7:**
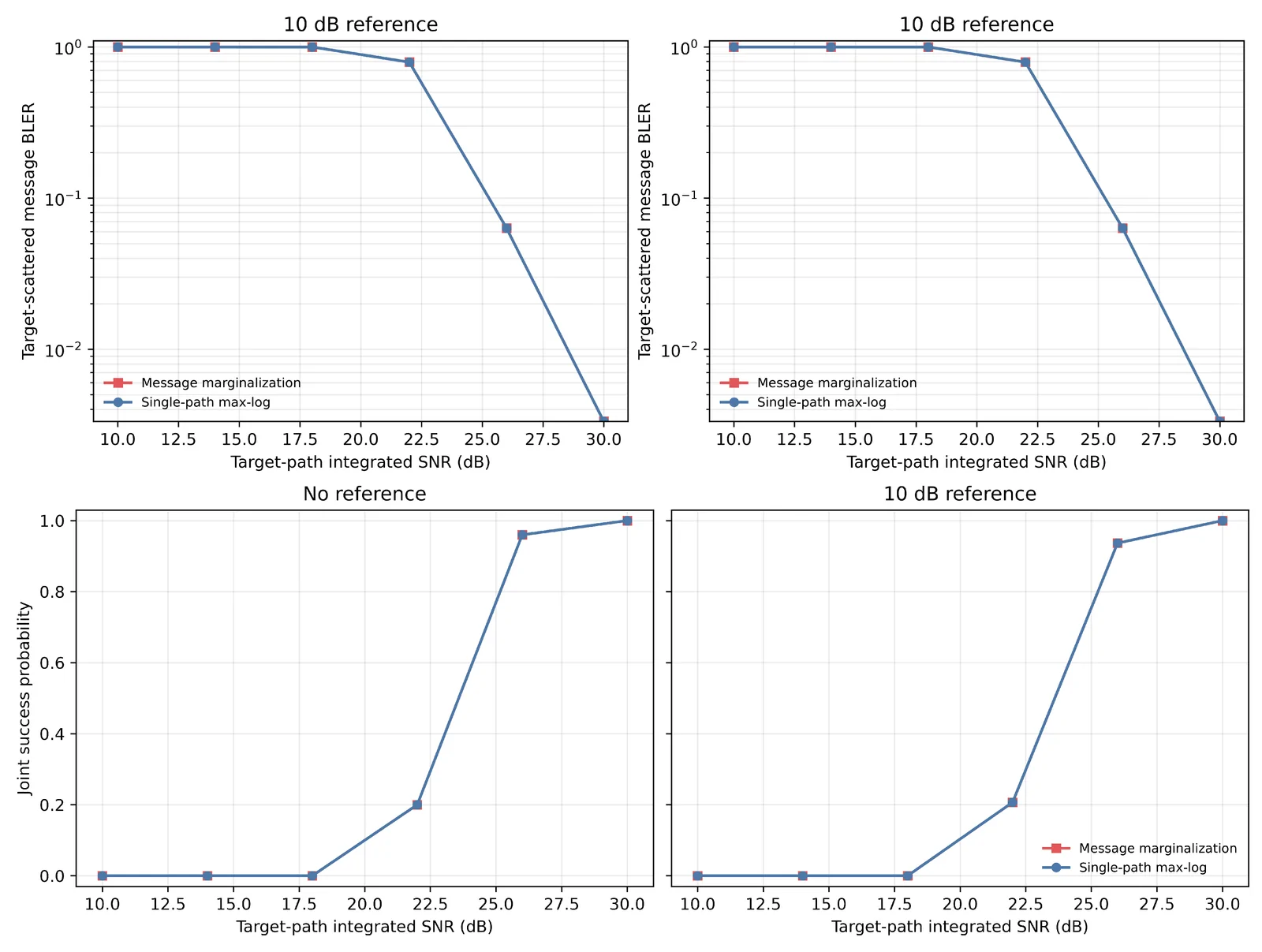
Full-frame target-scattered message BLER and joint-success probability for no-reference and 10 dB reference-path cases. All curves use the 46+2-symbol, 92-bit frame; zero observed BLER is plotted at 1/300.

Figure 6 and Figure 7 evaluate the complete 46+2-symbol, 92-bit frame with 16 states. Thresholds target a false-alarm probability of 0.01 and are calibrated independently for each receiver and reference condition. Detection approaches the Station B same-geometry known-message reference as target SNR increases. At a 10 dB reference SNR and a 14 dB target-path SNR, the marginal receiver attains a detection probability of 0.74 (95% Wilson interval 0.69–0.79), a delay RMSE of 0.42 samples (95% bootstrap interval 0.36–0.48), and a Doppler RMSE of 0.0054 cycles/sample (0.0042–0.0064). At a 26 dB target SNR, the message BLER is 0.040 (0.023–0.069) without a reference and 0.063 (0.041–0.097) with the 10 dB reference. Message-marginal and single-path max-log curves overlap for BLER and joint success in this experiment.

**Figure 8:**
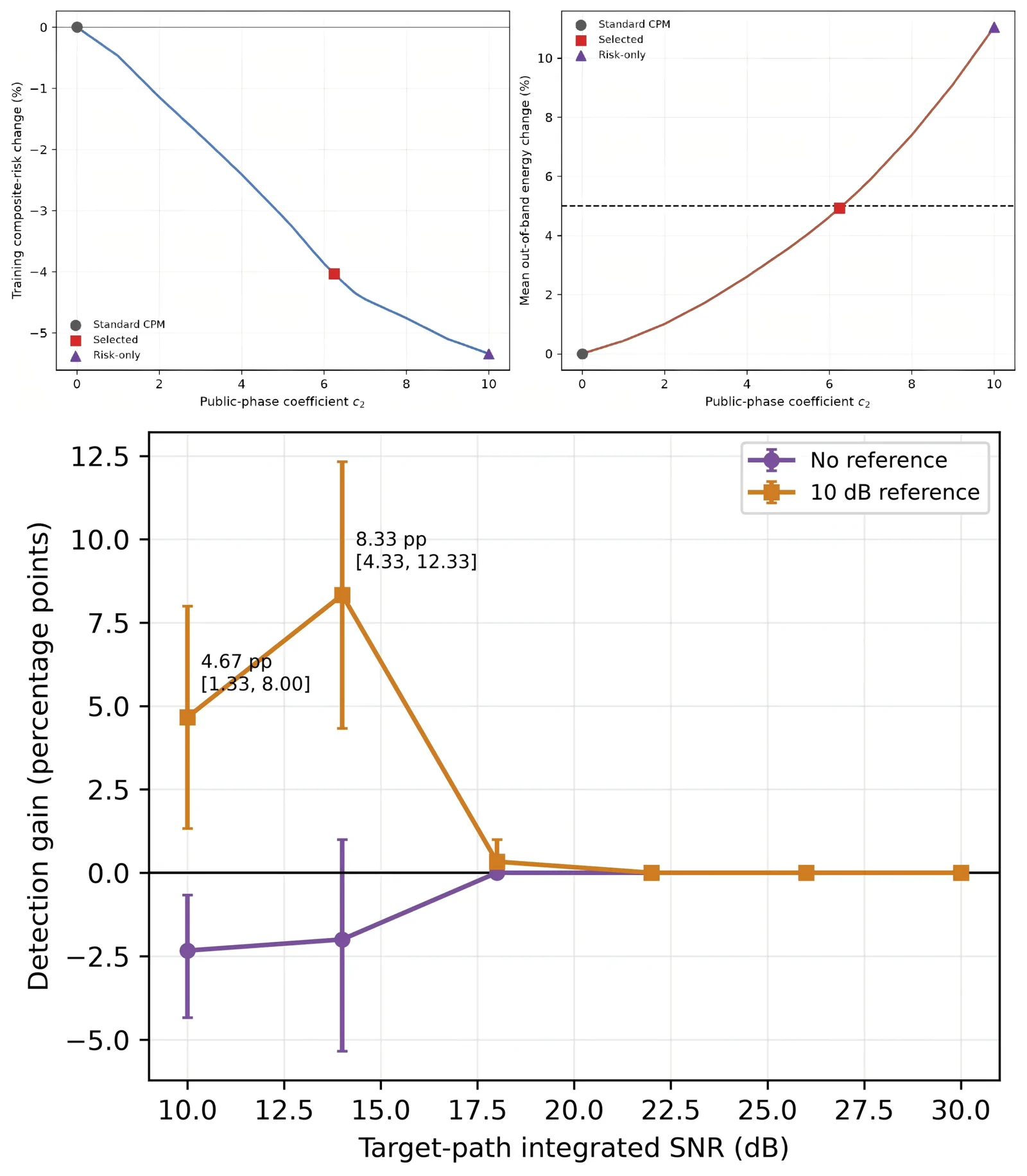
Ablation of the spectral constraint in common-phase selection and full-frame detection with message marginalization relative to single-path max-log reconstruction. Numeric labels give the paired 95% bootstrap intervals; the joint-success difference is zero at all tested points. Dashed lines indicate the zero-difference reference level.

Figure 8 gives the paired 92-bit receiver ablation and the spectral-constraint ablation. At a 10 dB reference-path SNR, message marginalization increases detection probability over single-path max-log reconstruction by 4.67 percentage points (95% paired bootstrap interval 1.33–8.00) at a 10 dB target-path SNR and by 8.33 percentage points (4.33–12.33) at 14 dB. The no-reference differences are not positive at these transition points, and the joint-success difference is zero at every tested SNR. The evidence therefore supports a localized detection benefit when the reference is informative but not decisive; it does not support the former broad joint-success claim.

The same figure also reports the spectral-constraint ablation. At $c_{2}=6.25$, training risk is reduced by 4.04% and out-of-band energy increases by 4.92%. At $c_{2}=10$, training risk is reduced by 5.34%, but out-of-band energy increases by 11.04% and violates the feasible-set definition.

**Figure 9:**
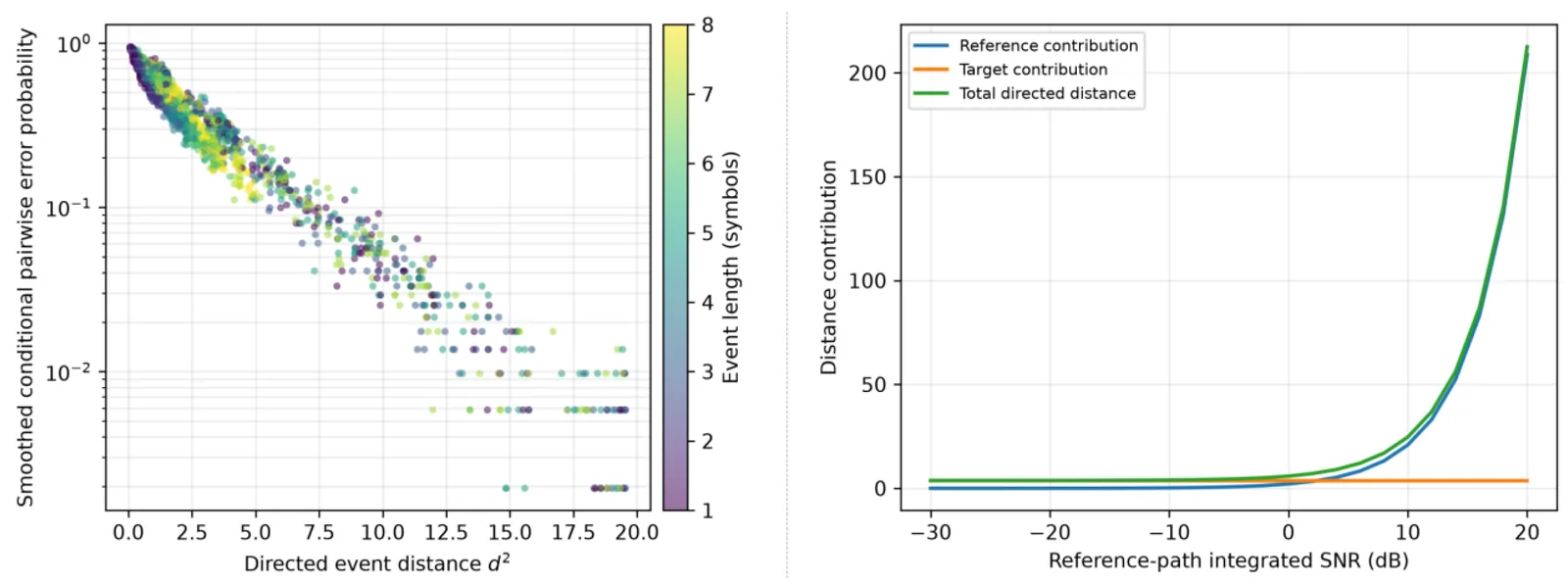
Directed conditional event distance showing empirical error ordering for 2048 ordered events and reference- and target-branch contributions as reference-path SNR varies.

The event-distance evaluation contains 2048 ordered events with lengths from one to eight symbols and 256 trials per event. The overall Spearman correlation between directed distance and empirical conditional pairwise error probability is −0.970, with a 95% bootstrap interval of −0.973 to −0.966. The correlations remain between −0.946 and −0.978 across no-reference, 0 dB-reference, and 6 dB-reference strata. Increasing the reference-path SNR raises only the reference contribution and leaves the target contribution unchanged in the conditionally independent model. Figure 9 therefore supports local error ordering and reference-limit continuity, not a global BLER bound.

### 5.4. Model Applicability and Scatterer-Model Boundary

The receiver likelihood assumes a conditionally Gaussian background and one dominant scattering center. Its applicability was assessed using target-absent and target-present records from the two-station coastal experiment described in Section 5.5. Because the propagation path crosses the sea, the background in these records includes sea-surface clutter and maritime multipath; array and oscillator effects, pointing offsets, and residual interstation timing and position errors are also present. The data set contains 24 target-absent and 24 target-present records. Twelve target-absent records are used only for empirical threshold calibration, and the remaining 12 are reserved for false-alarm evaluation. For normalized target-free power $P_{\mathrm{norm}}={\left|y\right|}^{2}/{E[|y|}^{2}]$, the circular complex-Gaussian model gives $\mathrm{Pr}(P_{\mathrm{norm}}>6)=0.00248$. Table 4 compares this reference with the empirical tails, cross-record covariance change, threshold outcomes, and range error.

At Stations A and B, the matched-filtered background has excess-kurtosis estimates of −0.11 and −0.20 and tail probabilities of 0.002 and 0.001, respectively, compared with the circular complex-Gaussian reference of 0.00248. The corresponding cross-record covariance differences are 0.72 and 0.69. At each station, both the nominal and empirically calibrated thresholds produce zero false alarms in 12 $H_{0}$ evaluation records and detect all 24 $H_{1}$ records. Secondary components consistent with sea-surface two-path propagation and target-path multipath are present in the records. These results show stable threshold decisions for the background observed during the coastal experiment and quantify the associated cross-record covariance variation.

The single-center boundary was examined using a separable pair, a nearby delay-Doppler component, four finite scattering centers, and finite Doppler spread. The same single-center processor and decision rule were retained. Because the strongest processed peak need not correspond to a predefined scattering center, Table 5 reports the error relative to the nearest constituent scattering center together with the offset of the energy centroid.

The separable pair yields three significant local peaks at Station A and three at Station B, while the nearby and few-center cases merge into one or two dominant responses. Across the four cases, the nearest-center range error remains below 14.97 m. The separable-pair energy centroid is displaced by 139.97 m at Station A and 298.01 m at Station B, and finite Doppler spread produces nearest-center Doppler errors of 30.38 and 81.72 Hz. Thus, the receiver continues to detect a dominant response, but close or distributed scattering can bias the reported parameters; component resolution and association require a model beyond the present scope.

### 5.5. Two-Station System-Level Experiment

**Figure 10:**
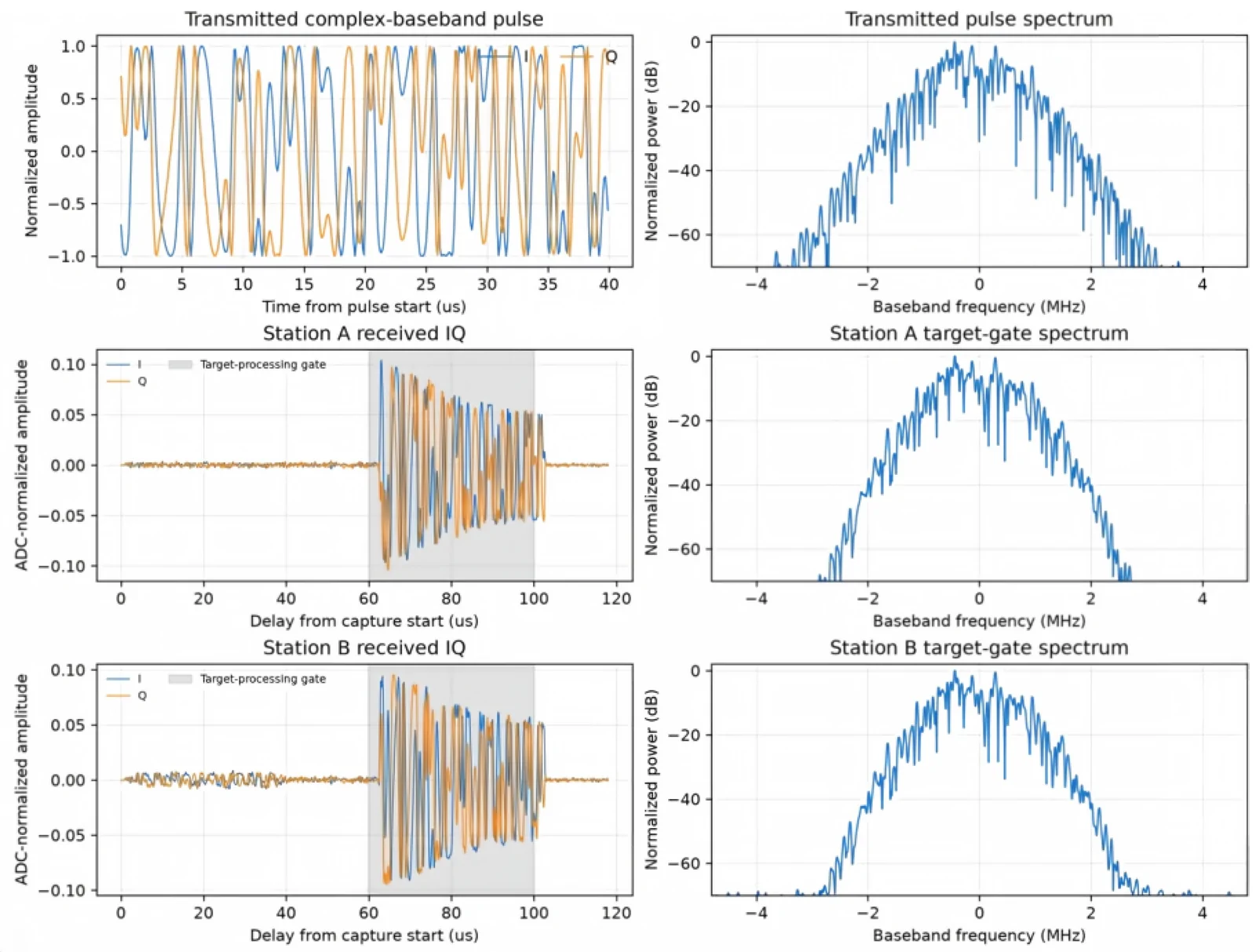
Transmitted and received complex-baseband records in the system-level experiment. Station A delay is referenced to the transmit epoch, and Station B delay is referenced to the direct-path arrival. The shaded interval denotes the target-processing gate, and dashed lines indicate its boundaries.

**Figure 11:**
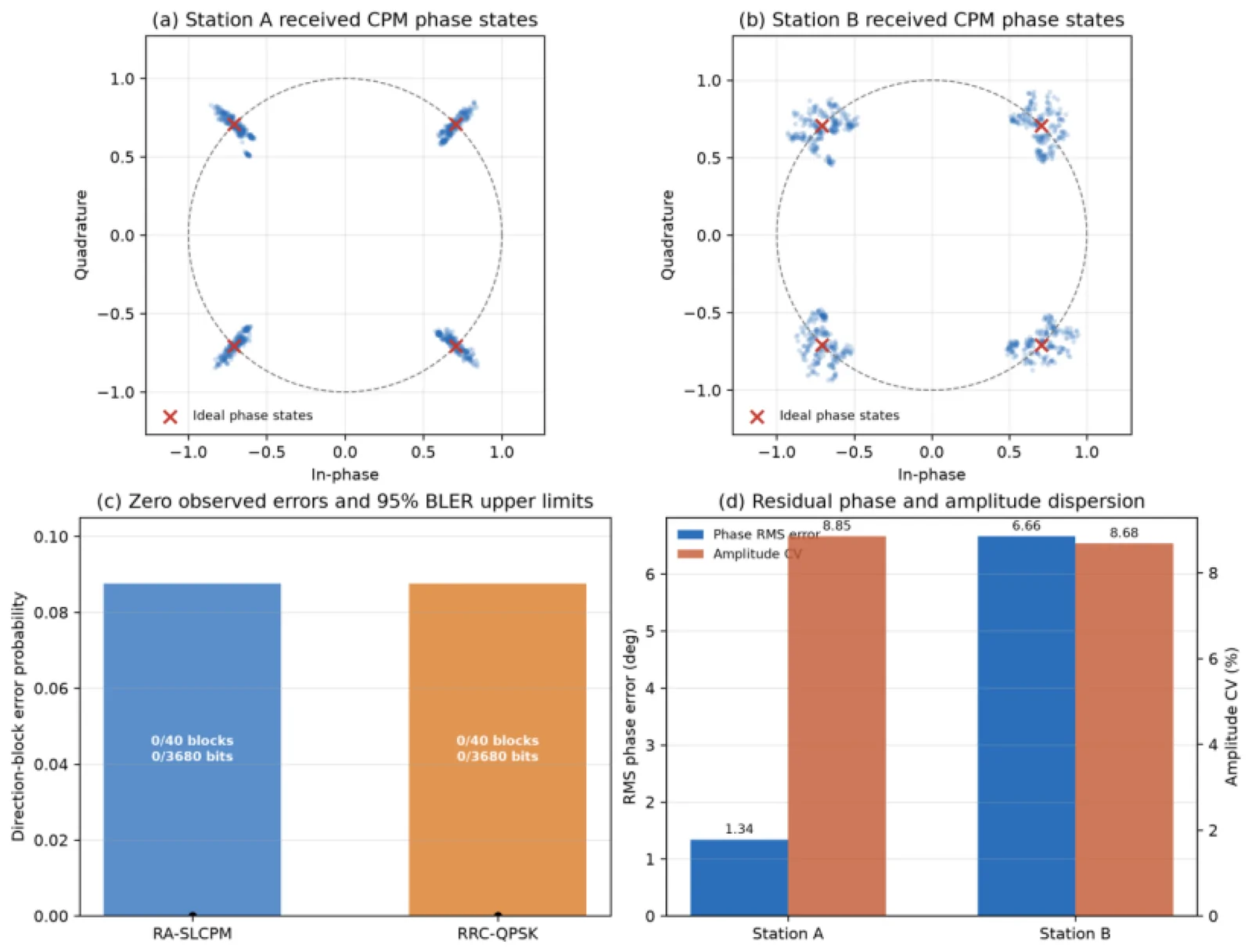
Direct-path communication results from the coastal system-level experiment: received CPM phase-state samples for the proposed waveform at (**a**) Station A and (**b**) Station B, where red crosses denote the ideal trellis phase states and no symbol-wise amplitude normalization is applied; (**c**) zero observed direction-block errors and 95% Wilson upper limits; and (**d**) phase-state RMS error and amplitude coefficient of variation at the two stations.

**Figure 12:**
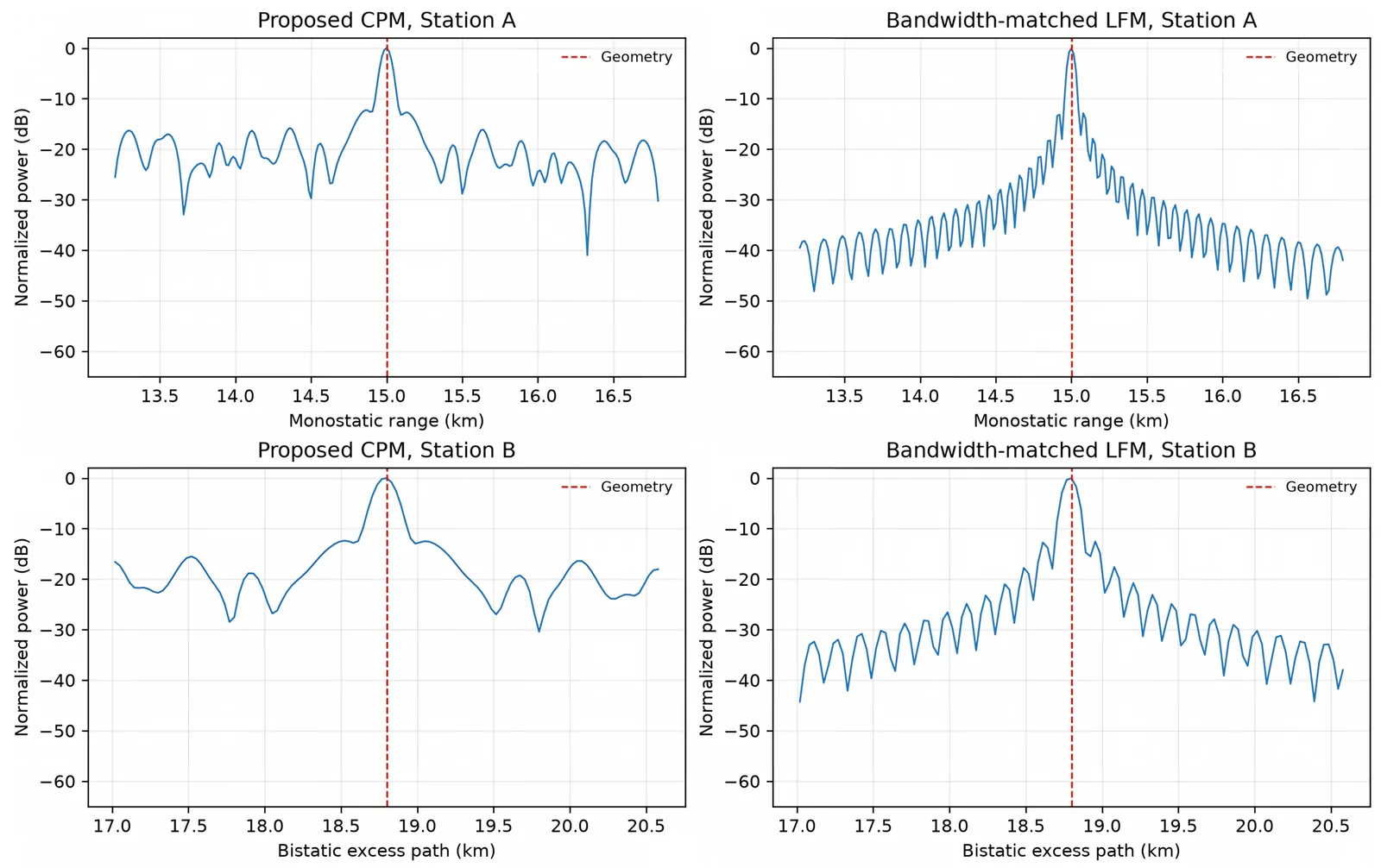
Pulse-compression results for the proposed CPM and bandwidth-matched LFM waveforms on the monostatic range at Station A and bistatic excess path at Station B.

**Figure 13:**
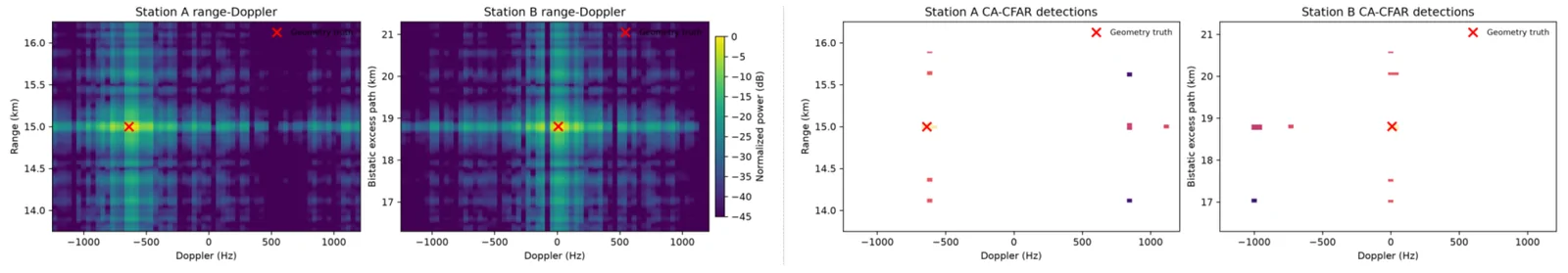
Proposed-CPM target processing on the monostatic-range and bistatic-excess-path coordinates, including range-Doppler maps and two-dimensional CA-CFAR decisions. Red crosses indicate the geometry-derived range-Doppler truth positions, colored points indicate CA-CFAR detections, and the background color scale represents normalized range-Doppler intensity.

The system-level experiment uses two fixed digital phased-array stations at coastal sites separated by approximately 11 km across an unobstructed maritime path. Each station operates with approximately 20 kW intrapulse transmit power, 40 dBi transmit and receive gains, and approximately $5^{\circ }$ azimuth and elevation 3 dB beamwidths. Satellite common-view time transfer maintains interstation timing throughout data acquisition, while the relative frequency offset of the local 100 MHz reference signals is continuously estimated and corrected. Surveyed station coordinates and the aircraft-borne GNSS trajectory provide the spatial reference for monostatic and bistatic parameter conversion. During the reported system-level experiment, the wind speed was approximately 6 m/s and the significant wave height was approximately 1.0 m. For direct-path communication, the beams point toward each other. For joint operation, both beams track an aircraft in the common-view region at approximately 15 km slant range, 2 km altitude, and 80 m/s speed; Station A forms the monostatic channel and Station B forms the bistatic channel. The model-applicability data comprise 24 $H_{0}$ and 24 $H_{1}$ records, while the direct-path communication comparison uses 20 independent payloads per waveform and two propagation directions. Figure 10, Figure 11, Figure 12 and Figure 13 present the joint-operation results, and the corresponding tables summarize the multi-record communication and radar evaluations.

Figure 10 presents the transmitted pulse and the Station A and Station B complex-baseband records before matched filtering. Station A delay is referenced to the transmit epoch, whereas Station B delay is referenced to the direct-path arrival. During joint operation, both array beams point toward the aircraft; the off-boresight direct path is consequently attenuated by the transmit and receive patterns, and the target-scattered component occupies the shaded processing gate in the Station B record. The time traces show one pulse, while the spectra average pulses 5–32.

Direct-path communication is evaluated using equal 40μs duration, 92-bit payload, normalized transmit energy, and receiver timing and gain estimation for the two waveforms. Each waveform uses 20 independent messages in two propagation directions, giving 3680 bit decisions and 40 direction blocks. The proposed CPM waveform and RRC-QPSK both produce zero bit errors and zero failed direction blocks. With no failures in 40 blocks, the 95% Wilson upper limit on direction BLER is 0.088 for each waveform. The proposed waveform therefore preserves direct-path payload recovery at the evaluated operating point, with no observed communication-error degradation relative to RRC-QPSK. Figure 11a,b show the received CPM phase-state samples for the proposed waveform after one common delay and one scalar complex-gain estimate per pulse. The deterministic radar phase sequence is removed, and the signal is sampled at the start of each symbol interval, where the CPM phase state is defined. No symbol-wise envelope normalization is applied. The radial dispersion retains the effects of receiver gain dynamics, propagation, front-end response, noise, and sampling error, while the angular dispersion includes residual phase, frequency, and timing errors. The four groups represent the h=1/2 trellis phase states rather than memoryless modulation symbols; payload recovery continues to use Viterbi sequence detection on the complete oversampled waveform. The amplitude coefficients of variation are 8.85% and 8.68%, and the phase-state RMS errors are $1.34^{\circ }$ and $6.66^{\circ }$, at Stations A and B, respectively. Figure 11c reports the observed block outcomes and their 95% upper limits, and Figure 11d summarizes the residual phase and amplitude dispersion.

In the repeated-message record, the aggregate of 5152 decisions is obtained as 2 directions × 28 evaluation pulses × 92 bits. Each direction repeats one 92-bit message across pulses, so these decisions are correlated repetitions rather than 5152 independent information bits. The independent-message results in Table 6 provide the CPM-QPSK communication comparison.

Figure 12 compares pulse compression of the proposed CPM and the bandwidth-matched LFM waveform under the same 40μs duration, pulse energy, average 99% occupied bandwidth, target geometry, and station-processing definitions. The A-station abscissa is monostatic slant range, whereas the B-station abscissa is bistatic excess path relative to the direct A-to-B reference. The geometry-derived target coordinates are approximately 15.00 and 18.80 km. The proposed CPM gives local peak-to-background ratios of 22.44 dB at Station A and 22.30 dB at Station B; the corresponding LFM values are 35.09 and 35.01 dB, differences of 12.65 and 12.71 dB. The higher LFM ratios and CFAR margins are consistent with its lower integrated sidelobe level. Both waveforms place the dominant peak in the geometry-derived target range cell. Because peak-to-background ratio depends on bandwidth, energy, geometry, background, and processing definition, cross-study numerical values are not pooled; Table 7 therefore uses the same-record LFM baseline.

Figure 13 presents the range-Doppler and CA-CFAR outputs for the proposed waveform using the station-specific coordinates of Figure 12. The estimated ranges differ from the GNSS-derived target coordinates by 0.09 m at Station A and 0.22 m at Station B. The Doppler errors are 11.81 and 7.03 Hz, respectively, within the resolution of the 32-pulse coherent interval. The target-cell CFAR margins are 3.41 and 3.01 dB. Additional detections outside the target neighborhood are consistent with clutter, multipath, and waveform-sidelobe components present in the records. The agreement between the detected peaks and the geometry-derived range-Doppler positions confirms target recovery at both stations.

On the target-scattered communication path, independent per-pulse decoding produces 121 errors in 2576 bit decisions and five erroneous blocks among 28 evaluation pulses. Rank-one repeated-message combining followed by finite-state sequence decoding recovers all 92 unique message bits. This result confirms operation of the B1 combining rule at the evaluated system-level operating point. Because only one 92-bit word is recovered, the zero-error outcome is a functional result rather than a low-BER estimate. The corresponding new-information rate is 7.19 kbit/s.

## 6. Discussion

The results distinguish waveform selection from receiver processing. Common-phase selection modestly changes the distribution of message-dependent ambiguity sidelobes while preserving the imposed GMI and spectral constraints; the validation interval for the CVaR component includes zero. The full-frame likelihood study instead identifies two 10 dB-reference transition points at which message marginalization improves detection over max-log reconstruction. Because BLER and joint-success differences are zero in the same study, the receiver conclusion is confined to detection under those conditions.

Reference-assisted factorization requires a statistic whose target-independent interpretation is unchanged under both target hypotheses. Correlated reference and target statistics must be represented by their conditional joint density. Without a reference, the target-scattered message enters only under the target-present hypothesis and is marginalized jointly with target parameters. The resulting continuous dependence on reference-path quality is specific to this likelihood model; related auxiliary-link reliability transitions have also been observed in cooperative communication architectures [44], although their signal and channel models differ.

The likelihood construction can accommodate additional receiving branches when they share the same CPM message state graph and their synchronization, geometry, and covariance models are known or included in the search. Independent branches add log branch metrics, whereas correlated observations require the corresponding joint covariance; branch-metric cost grows approximately linearly with receiver count without increasing the CPM state count. The present validation is limited to the two-station monostatic/bistatic configuration and one dominant scattering center.

Strict envelope constancy before the fixed array weights permits near-saturated per-element transmission. The evaluated CPM frame, however, requires two termination symbols and approximately twice the occupied bandwidth of RRC-QPSK at equal duration and payload. Its common-phase adjustment is small at the selected spectral tolerance, and LFM gives stronger conventional pulse-compression background suppression. The proposed signal is consequently suited to radar-centric constant-envelope data transmission with joint reception; it is not intended to supersede LFM in every radar metric or QPSK in spectral efficiency.

The numerical and system-level evaluations provide complementary evidence for waveform and receiver performance. Receiver curves use independent threshold-calibration and evaluation samples on the complete 92-bit frame. The coastal records contain sea-clutter and multipath components, propagation loss, array and oscillator effects, pointing offsets, and residual interstation errors, and they yield consistent decisions under nominal and empirically calibrated thresholds. The finite-scatterer cases show that the single-center processor detects a dominant response, although nearby, distributed, or Doppler-spread components can bias the reported parameters. These results support the single-center receiver when a dominant return is present and identify the expected estimation effects of distributed scattering. The repeated target-scattered message result demonstrates the combining procedure, while independent-message direct-path statistics provide the communication comparison.

## 7. Conclusions

A strictly constant-envelope pulsed CPM waveform and a likelihood-consistent joint target-message receiver were developed for monostatic and bistatic JRC with an available, degraded, or absent target-independent reference path. The 16-state recursion evaluates the complete 92-bit message marginal and is numerically verified against direct enumeration on a short unit frame. Full-frame results show statistically supported detection gains over max-log reconstruction at two finite-reference transition points, without a corresponding joint-success gain. The directed event distance orders 2048 evaluated pairwise events and remains continuous as the reference contribution vanishes. Common-phase selection provides a modest secondary adjustment under the spectral and GMI constraints. System-level experiments demonstrate direct-path payload recovery without observed degradation relative to RRC-QPSK, geometry-consistent monostatic and bistatic processing, and target-scattered repeated-message recovery. The coastal-background and finite-scatterer evaluations characterize threshold behavior and delimit the single-scattering-center model. The demonstrated scope is limited to the two-station monostatic/bistatic configuration; extension to additional receiver branches remains a methodological generalization rather than a validated result.

## Figures and Tables

**Table 1:** Likelihood-consistent joint target-message receiver at Station B.

Stage	Operation
Input	Reference and target-channel observations; CPM state graph; target-parameter grid; background statistics; detection threshold γ.
Output	Log-likelihood ratio, target decision, target-parameter estimate, message posterior or LLRs, and message estimate.
1	Construct the reachable CPM state graph and the delayed-Doppler branch templates.
2	When a target-independent reference is available, calculate its branch metrics; otherwise initialize the branches from the message prior.
3	Evaluate the target-absent likelihood with the target-dependent component removed.
4	For each target-parameter and framewise complex-gain candidate, combine the reference or prior term with the target-channel branch metric.
5	Run the log-domain forward recursion at the fixed candidate and obtain the message-marginal target-present likelihood.
6	Maximize the target-present likelihood over the outer parameter grid and form the log-likelihood ratio.
7	Compare the log-likelihood ratio with the independently calibrated threshold γ.
8	After a target-present decision, run the backward recursion and maximum-path traceback at the selected parameter point to obtain message posteriors, LLRs, and the message estimate.

**Table 2:** Principal evaluation conditions.

Category	Setting
CPM	h=0.5; length-two raised-cosine frequency pulse; 16 states
Pulse	40μs; 10% duty cycle; 32 pulses per coherent interval
Payload	46 information symbols + 2 termination symbols; 92 bits/pulse
Waveform design	256 training messages; 2048 independent validation messages; GMI evaluated at 4 dB
Receiver grid	Reference absent or 10 dB; target SNR 10–30 dB; 2 delays × 3 Dopplers × 57 complex-gain candidates
Monte Carlo	1000 $H_{0}$ calibration + 1000 independent $H_{0}$ evaluation frames per reference condition; 300 $H_{1}$ frames per SNR point
Intervals	95% Wilson intervals for probabilities; paired frame bootstrap for differences; frame bootstrap for RMSE
Comparison controls	Radar baseline: LFM matched to the proposed CPM waveform in 40μs duration, pulse energy, and average 99% occupied bandwidth. Communication baseline: RRC-QPSK matched in 40μs duration, 92-bit payload, and normalized pulse energy, with 0.25 root-raised-cosine roll-off.
System-level records	Coastal system-level experiment; approximately 6 m/s wind speed and 1.0 m significant wave height during the reported test; 24 $H_{0}$ + 24 $H_{1}$ records; 20 independent messages per waveform
Geometry	Two arrays about 11 km apart; common-view aircraft about 15 km from Station A; maritime multipath and sea-clutter background

**Table 3:** State-mass approximation for the 92-bit, 16-state receiver at the fixed validation point.

Receiver	Avg./Max. Retained States (%)	95th-Percentile |Δℓ|: $H_{0}/H_{1}$	$\Delta P_{d}/\Delta \mathrm{BLER}$	Median Time (ms/Pulse)
Complete (ϵ=0)	100/100	0/0	0/0	256.4
$\epsilon =10^{-4}$	54.3/100	0.0010/0.0004	0/0	303.7
$\epsilon =10^{-3}$	47.9/100	0.0061/0.0036	0/0	304.1
$\epsilon =10^{-2}$	39.9/100	5.31/6.20	0/+0.075	302.8

**Table 4:** Model-applicability results for the coastal system-level experiment. Threshold columns report false alarms among 12 independent $H_{0}$ evaluation records and detections among 24 $H_{1}$ records at each station; the empirical threshold is calibrated on a separate set of 12 $H_{0}$ records.

Condition/Station	$P(P_{\mathrm{norm}}>6)$	Covariance Change	Nominal: FA; D	Empirical: FA; D	Median Range Error (m)
Mild/A	0.001	0.39	0/12; 24/24	0/12; 24/24	2.47
Mild/B	0.002	0.45	0/12; 24/24	0/12; 24/24	6.23
Coastal/A	0.002	0.72	0/12; 24/24	0/12; 24/24	3.16
Coastal/B	0.001	0.69	0/12; 24/24	0/12; 24/24	6.46
Rough/A	0.005	0.49	0/12; 24/24	0/12; 24/24	3.47
Rough/B	0.003	0.46	0/12; 23/24	0/12; 23/24	10.06

**Table 5:** Boundary evaluation of the single-scattering-center receiver.

Scatterer Case	Centers	Resolved Peaks A/B	Nearest Range Error A/B (m)	Nearest Doppler Error A/B (Hz)	Range-Centroid Offset A/B (m)
Separable pair	2	3/3	0.23/0.03	67.68/82.41	139.97/298.01
Nearby component	2	2/2	7.66/14.97	55.57/37.12	0.17/4.24
Few-center target	4	1/2	12.06/0.08	16.16/28.57	23.95/35.56
Doppler spread	3	3/2	4.34/5.50	30.38/81.72	18.97/49.21

**Table 6:** Matched direct-path communication results with independent 92-bit messages.

Environment	Independent Messages/Method	Bit Decisions/Method	Proposed CPM Errors; Failed Blocks	RRC-QPSK Errors; Failed Blocks
Coastal system-level experiment	20	3680	0; 0/40	0; 0/40

**Table 7:** Representative radar-processing results for the joint-operation record in Figure 12 and Figure 13.

Waveform/Station	Peak/Background (dB)	Range Error (m)	Doppler Error (Hz)	CFAR Margin (dB)	Target Cell
Proposed CPM/A	22.44	0.09	11.81	3.41	Detected
Proposed CPM/B	22.30	0.22	7.03	3.01	Detected
LFM/A	35.09	0.09	11.81	6.57	Detected
LFM/B	35.01	0.22	7.03	5.91	Detected

## Data Availability

The waveform-generation, receiver-processing, and numerical-evaluation code and the data required to reproduce the reported results will be made available with the published article or in a public repository.
